# A Rare Hematopoietic Stem Cell‐Derived Megakaryocyte Progenitor Accumulates via Enhanced Survival and Contributes to Exacerbated Thrombopoiesis Upon Aging

**DOI:** 10.1111/acel.70221

**Published:** 2025-09-23

**Authors:** Bryce A. Manso, Paloma Medina, Stephanie Smith‐Berdan, Alessandra Rodriguez y Baena, Elmira Bachinsky, Lydia Mok, Angela Deguzman, Sarah Beth Avila, Connor Van Voorhis, Saran Chattopadhyaya, Marcel G. E. Rommel, Jenna Myers, Vanessa D. Jönsson, E. Camilla Forsberg

**Affiliations:** ^1^ Institute for the Biology of Stem Cells, University of California Santa Cruz California USA; ^2^ Biomolecular Engineering Baskin Engineering, University of California Santa Cruz California USA; ^3^ Program in Biomedical Science and Engineering University of California Santa Cruz California USA; ^4^ Genomics Institute University of California Santa Cruz California USA

**Keywords:** aging, blood platelets, hematopoiesis, hematopoietic stem cells, megakaryocyte progenitor cells

## Abstract

Distinct routes of cellular production from hematopoietic stem cells (HSCs) have defined our current view of hematopoiesis. Recently, we and others have challenged classical views of platelet generation, demonstrating that megakaryocyte progenitors (MkPs) and ultimately platelets can be specified via an alternate and additive route of HSC‐direct specification specifically during aging. This “shortcut” pathway generates hyperactive platelets likely to contribute to age‐related platelet‐mediated morbidities. Here, we used single‐cell RNA/CITEseq to demonstrate that these age‐unique, noncanonical (nc)MkPs can be prospectively defined and experimentally isolated from wild‐type mice. Surprisingly, this revealed that a rare population of ncMkPs also exists in young mice. Young and aged ncMkPs are functionally distinct from each other and from their canonical (c)MkP counterparts, with aged ncMkPs paradoxically and uniquely exhibiting enhanced survival and platelet generation capacity. We further demonstrate that aged HSCs generate significantly more ncMkPs than their younger counterparts, yet this is accomplished without strict clonal restriction. Together, these findings reveal significant phenotypic, functional, and aging‐dependent heterogeneity among the MkP pool and uncover unique features of megakaryopoiesis throughout life, potentially offering cellular and molecular targets for the mitigation of age‐related adverse thrombotic events.

## Graphical abstract

Manso et al. developed strategies for isolating and testing the functional mechanisms regulating age‐specific megakaryocyte progenitors derived from hematopoietic stem cells.

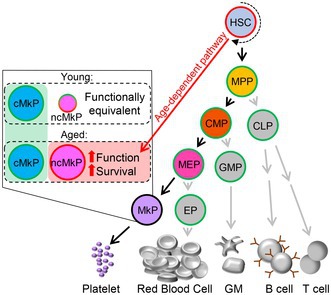

**Abbreviations**
BM: bone marrow
CITE: Cellular Indexing of Transcriptomes and Epitopes
CLP: common lymphoid progenitor
cMkP: canonical megakaryocyte progenitor
CMP: common myeloid progenitor
EP: erythroid progenitor
FACS: fluorescence activated cell sorting
GM: granulocyte/monocyte
GMP: granulocyte monocyte progenitor
HSC: hematopoietic stem cell
MEP: megakaryocyte erythroid progenitor
MFI: median fluorescence intensity
MkP: megakaryocyte progenitor
MPP: multipotent progenitor
ncMkP: noncanonical megakaryocyte progenitor
RBC: red blood cell
scRNA/CITEseq: single‐cell RNA and CITE sequencing
Tom: tdTomato

## 1 Introduction

Aging is associated with remarkable changes to hematopoiesis, resulting in altered blood cell output and function (Manso et al. 2024; Poscablo et al. 2024; de Haan and Lazare 2018; Geiger et al. 2013; Rossi et al. 2008; Paul et al. 2016). The aging population has an increased risk of developing a host of disorders including cancer (White et al. 2014), arthritis (Loeser 2017), diabetes (Kalyani et al. 2017), neurodegeneration (Hou et al. 2019), and cardiovascular (Rodgers et al. 2019) diseases; maladies associated with age‐related alterations in hematopoiesis (Chung and Park 2017; Jaiswal and Ebert 2019; Cook et al. 2019; Nikolich‐Zugich 2018). One major age‐related health concern is platelet‐mediated contributions to cardiovascular disease (Le Blanc and Lordkipanidze 2019). As such, understanding the cause and consequence of hematopoietic aging is imperative to improve healthy aging. Platelets, anucleate cell fragments, are continuously produced in the bone marrow (BM) by hematopoietic stem and progenitor cells (HSPCs) and function by mediating blood clotting, wound healing, and immune responses (Koupenova et al. 2018; Nurden 2011; Holinstat 2017). With aging, alterations in the number of platelets combined with a lower threshold of activation collectively contribute to platelet‐mediated diseases common in the aging population (Le Blanc and Lordkipanidze 2019; Davizon‐Castillo et al. 2019; Dayal et al. 2013; Gleerup and Winther 1995; Johnson et al. 1975; Jones 2016; Martin et al. 2024; Mohebali et al. 2014; Mojadidi et al. 2014; Montenont et al. 2019; Moulis et al. 2014; Yang et al. 2016). Despite a plethora of platelet‐targeting drugs, cardiovascular disease remains the leading cause of death, illustrating the urgent need for novel insight into aging‐specific thrombopoiesis.

**FIGURE 1:**
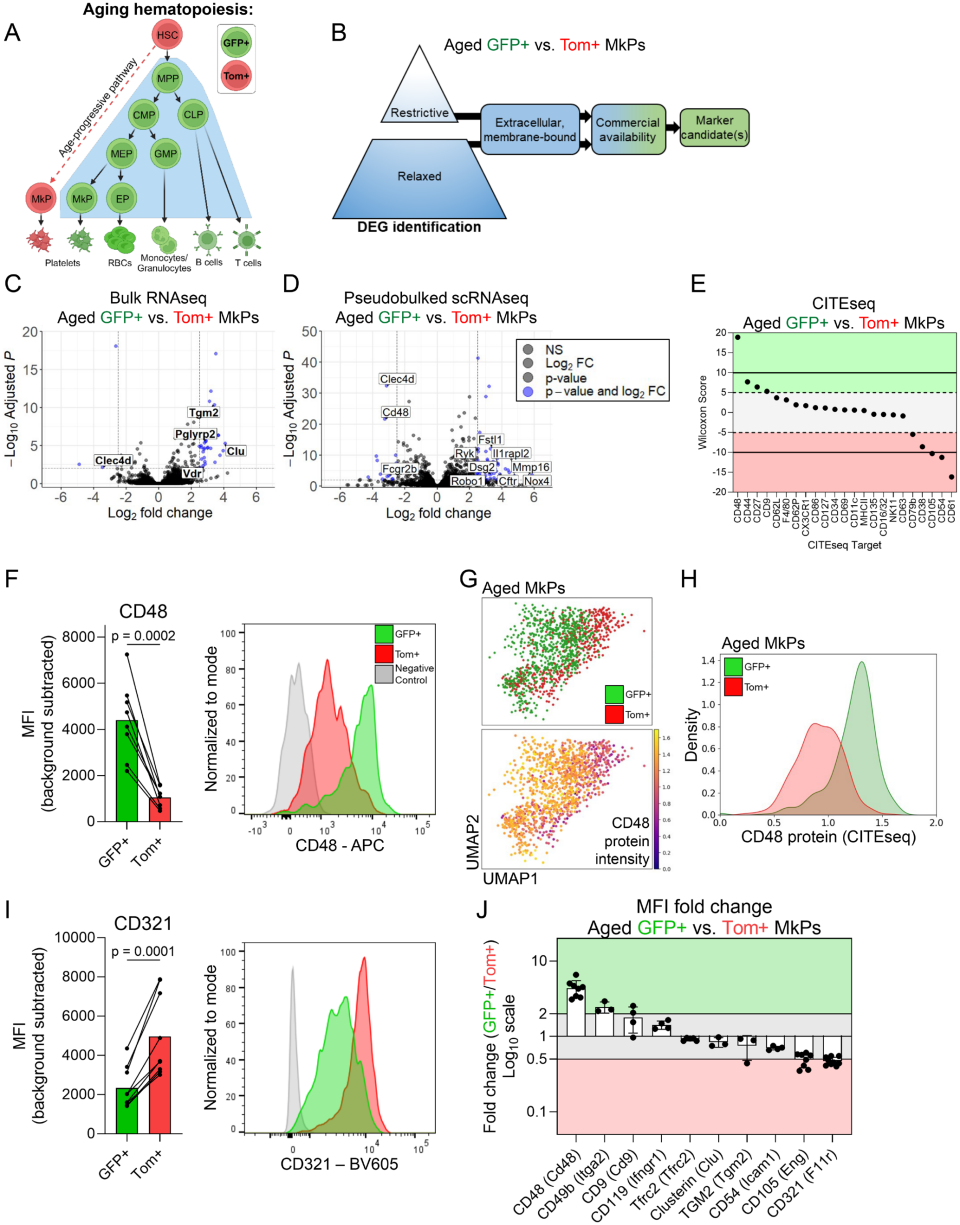
Aged GFP+ and Tom+ MkPs possess unique transcriptomic and proteomic profiles. (A) Schematic of hematopoiesis in aging FlkSwitch mice (Manso et al. 2024; Poscablo et al. 2024; Boyer et al. 2011). In this model, HSCs express the tdTomato (Tom) transgene. Upon differentiation to multipotent progenitors (MPPs), recombination mediated by *Flk2*‐ (*Flt3*)‐Cre deletes the Tom transgene and allows irreversible expression of GFP+. Thus, all downstream progenitors and mature cells remain GFP+ for life. Canonical megakaryocytic specification then progresses through a series of increasingly restricted myeloid progenitors including common myeloid progenitors (CMPs), megakaryocyte‐erythroid progenitors (MEPs), and megakaryocyte progenitors (MkPs). During aging, a unique and additive population of Tom‐expressing MkPs and platelets arises directly from HSCs (Poscablo et al. 2024). Shaded area represents the view of canonical hematopoiesis. Red and green colors represent Tom and GFP expression, respectively. CLP, common lymphoid progenitor; EP, erythroid progenitor; GMP, granulocyte monocyte progenitor. (B) Schematic of candidate marker determination from bulk RNAseq and scRNA/CITEseq. Volcano plots of (C) bulk and (D) single cell pseudobulked RNAseq data comparing aged FlkSwitch GFP+ and Tom+ MkPs from Poscablo et al. 2024. Restrictive thresholds are adjusted *p* value < 0.01 and absolute log_2_ fold change ≥ 2.5, indicated by blue points. Labeled genes indicate those predicted to encode cell surface proteins that have commercially available flow cytometry compatible antibodies. (C) 8120 total genes and (D) 11,570 total genes plotted. (E) CITEseq analysis of aged GFP+ and Tom+ MkPs ran simultaneously with scRNAseq. Dashed lines indicate Wilcoxon Score cutoffs (restrictive cutoffs at 10 and −10, black lines, and relaxed thresholds at 5 and −5, blue lines). (F) Flow cytometry analysis of CD48 expression (background subtracted median fluorescent intensity [MFI]) among aged FlkSwitch GFP+ and Tom+ MkPs. Each point represents a single mouse, with lines connecting cells from the same animal. Example flow cytometry histogram indicating the fluorescence minus one (FMO) control (gray), GFP+ MkPs (green), and Tom+ MkPs (red). *n* = 8 mice across five independent experiments. (G) Uniform Manifold Approximation and Projection (UMAP) of aged GFP+ and Tom+ MkPs from FlkSwitch mice. Top panel indicates MkPs classified as either GFP+ or Tom+ as in Poscablo et al. 2024. Bottom panel demonstrates CD48 CITEseq protein expression intensity across the same cells. (H) Histogram display of CD48 CITEseq protein expression across aged GFP+ and Tom+ MkPs from the scRNAseq data in (G) GFP+ MkPs (green) and Tom+ MkPs (red). (I) Flow cytometry analysis of CD321 expression (background subtracted MFI) among aged FlkSwitch GFP+ and Tom+ MkPs. Each point represents a single mouse with lines connecting cells from the same animal. Example flow cytometry histogram indicating the fluorescence minus one (FMO) control (gray), GFP+ MkPs (green), and Tom+ MkPs (red). *n* = 9 mice across six independent experiments. (J) Summary of cell surface candidates as a measure of fold change in relative protein expression (gene names indicated in parentheses). Horizontal lines indicate two‐fold change threshold (on a log_10_ *y*‐axis scale). Shaded areas indicate if markers are more prevalent on GFP+ (green) or Tom+ (red) aged FlkSwitch MkPs. See also Figure S1. Data for CD119 and CD105 from Poscablo et al. 2024 with additional CD105 replicates.

Classically, platelets arise via successive differentiation of hematopoietic stem cells (HSCs) through increasingly restrictive progenitor cell states, including specification into unilineage megakaryocyte progenitors (MkPs, Figure 1A, shaded area) that ultimately mature into megakaryocytes which release platelets into the blood (megakaryopoiesis) (Manso et al. 2024; Poscablo et al. 2024; Machlus and Italiano Jr. 2013). Recent advances in understanding platelet generation have given rise to new hypotheses of alternative routes of young adult, steady‐state platelet specification (reviewed here (Manso et al. 2024)). Notably, we have definitively demonstrated that an additional and progressive platelet generation pathway is uniquely pronounced during aging (Poscablo et al. 2024) (Figure 1A, dashed line) with minimal contribution in young mice, as suggested by some (Carrelha et al. 2024), but not all (Li et al. 2024; Morcos et al. 2022), recent reports. Using Flk2‐Cre mT/mG (“FlkSwitch”) lineage tracing mice (Figure S1A) (Poscablo et al. 2024; Boyer et al. 2011, 2012; Hoeffel et al. 2015; Epelman et al. 2014; Hashimoto et al. 2013; Beaudin et al. 2016; Cool et al. 2020; Leung et al. 2019; Worthington et al. 2022), we discovered that this additional path arises directly from aged HSCs, bypassing canonical intermediate progenitors, generating an additional pool of MkPs with unique molecular and functional properties that coexist with classically derived MkPs (Poscablo et al. 2024). Importantly, MkPs generated via this additional HSC‐direct (tdTomato [Tom]+) pathway give rise to platelets with hyperactive function, likely serving as a key contributor to the onset and progression of platelet‐mediated adverse health events that drastically increase upon aging. Thus, platelet precursor cells may be key to understanding and controlling downstream platelet function and for identifying novel therapeutic interventions.

Here, we pursued the molecular features and functional heterogeneity among MkPs that remain largely unexplored, particularly during aging. We sought to determine if the age‐unique Tom+ MkPs could be prospectively identified and isolated without the need of genetic reporters, thereby tackling the seeming inconsistencies across recent studies (Poscablo et al. 2024, 2021; Carrelha et al. 2024; Li et al. 2024; Morcos et al. 2022; Boyer et al. 2011) and facilitating future age‐specific MkP identification in alternative genetic models. We explored if young MkPs can be phenotypically stratified similar to their aged counterparts, assessed if young MkP subpopulations also possess unique functional capacities, and if enhanced aged MkP function is rooted in key molecular alterations. We further explore if age‐related HSC clonal restriction produces specific MkP subsets and if removal from the aged BM environment influences the route(s) of MkP output of HSCs.

## 2 Results

### 2.1 Aged MkPs Exhibit Distinct Molecular and Phenotypic Heterogeneity

Age‐progressive, Tom+ MkPs from old FlkSwitch mice exhibit significant molecular, phenotypic, and functional differences compared to their GFP+ canonical counterparts (Poscablo et al. 2024). Thus, we began by profiling cell surface protein candidates likely to be differentially expressed between aged (20–24 month) Tom+ and GFP+ MkPs, with the initial goal of enriching these populations without the need of the FlkSwitch lineage tracing model. To identify candidates, we took a top‐down approach using multimodal data of aged GFP+ and Tom+ MkPs from FlkSwitch mice (Figure 1B). We performed in‐depth analysis of our published (Poscablo et al. 2024, 2021) bulk RNA sequencing (RNAseq) and single cell RNAseq (scRNAseq) data for differentially expressed genes (DEGs) using restrictive statistical cutoffs (Figure 1C,D, Table S1). Candidate selection among DEGs was further narrowed to those predicted to code for membrane‐bound, extracellular proteins. As a major goal was to prospectively isolate aged MkP subsets without using FlkSwitch (or other genetically modified) mice, we further limited our inclusion criteria to DEGs with commonly used and commercially available flow cytometry antibodies, excluding those used to phenotypically define MkPs (Lin^low^cKit+Sca1‐CD150+CD41+, Figure 1B and Figure S11A,B). This approach identified five candidates from bulk RNAseq and 11 from scRNAseq (Figure 1C,D, Table S1). Simultaneous with scRNAseq, we generated single cell proteomics via Cellular Indexing of Transcriptomes and Epitopes sequencing (scCITEseq) data of HSPCs from the same FlkSwitch mice. Four candidates were identified using a restrictive cutoff (Figure 1E, solid lines, Table S1).

We selected candidates to test with more extreme differences among any of the restrictive data sets, reasoning that those are most likely to have detectable differences in protein expression by flow cytometry. We initially tested protein expression for the *Tgm2* (transglutaminase 2), *Clu* (clusterin/APO‐J), and *Icam1* (CD54) genes by flow cytometry, comparing aged GFP+ and Tom+ MkPs from FlkSwitch mice (Figure S1B–D). Of these, only CD54 had significant differences in relative protein levels, yet the magnitude of separation was not compatible with efficient flow cytometry‐based cell sorting.

We also tested *Cd48* (CD48/SLAMF2) as it was identified in the restrictive scRNA/CITEseq data sets. Further, differential CD48 expression was recently reported among young adult MkPs and hypothesized to identify MkPs generated via different routes (Carrelha et al. 2024; Li et al. 2024; Morcos et al. 2022). CD48 demonstrated robust expression among aged GFP+ MkPs with a high degree of separation from aged Tom+ MkPs (Figure 1F). By way of confirmation, overlaying CD48 protein expression (by scCITEseq) on our scRNAseq data further illustrates its differential expression between aged GFP+ and Tom+ MkP subpopulations (Figure 1G,H). Thus, CD48 is a likely candidate for separating the GFP+ and Tom+ MkP subpopulations in old mice.

Flow cytometry cell categorization, and especially isolation, performs optimally when populations are identified using more than one marker. Thus, we next sought to identify an additional cell surface candidate to pair with CD48 to enrich for GFP+ and Tom+ aged MkPs. Given that strong statistical and log_2_ fold change differences among DEGs do not necessarily reflect detectable differences at the protein level (Figure S1B–D), we reasoned that relaxing our inclusion criteria and selecting for DEGs present across both RNAseq datasets would represent likely candidate markers (Figure S1E,F; Table S1). We found 23 shared genes between bulk and scRNAseq (Table S2). From these, we selected *Tfr2* (transferrin receptor 2/TFRC2), *Cd9* (CD9/TSPAN29), and *F11r* (CD321/JAM1/JAMA) to test based on the commercial availability of antibodies against their protein products. A relaxation in the scCITEseq criteria similarly confirmed CD9 as a potential candidate (Figure 1E dashed lines, Table S1). We also tested CD49b as it was recently reported to have differential expression among young adult MkPs (Carrelha et al. 2024) and was identified in our relaxed scRNAseq data set (Table S1). Flow cytometry assessment of TFRC2, CD9, and CD49b between aged GFP+ and Tom+ MkPs revealed no clear distinction (Figure S1G–I). However, CD321 staining was consistently elevated among aged Tom+ MkPs and distinct from GFP+ MkPs (Figure 1I), representing a second potential candidate. *Eng* (endoglin/CD105) and *Ifngr1* (CD119) were also identified as DEGs, which we previously demonstrated have significant differential protein expression (Poscablo et al. 2024).

To determine which candidates are most likely to enable efficient identification and prospective isolation of aged GFP+ and Tom+ MkPs, we evaluated the fold change median fluorescent intensity (MFI) between them for each marker tested (Figure 1J). We reasoned that any usable marker would exhibit robust and reproducible staining patterns, have consistently ample separation compatible with efficient flow cytometry isolation approaches, and that separation would be a minimum of a two‐fold MFI difference. Although a potential candidate, CD105 proved to be unreliable due to variability in separation magnitude between aged Tom+ and GFP+ MkPs (Figure 1J). Thus, CD48 and CD321 were the only two candidates satisfying all criteria. Importantly, it is advantageous to have one marker that positively selects for GFP+ (CD48) and Tom+ (CD321) aged MkPs.

### 2.2 CD48 and CD321 Coordinately Enrich for Aged Tom + MkPs

**FIGURE 2:**
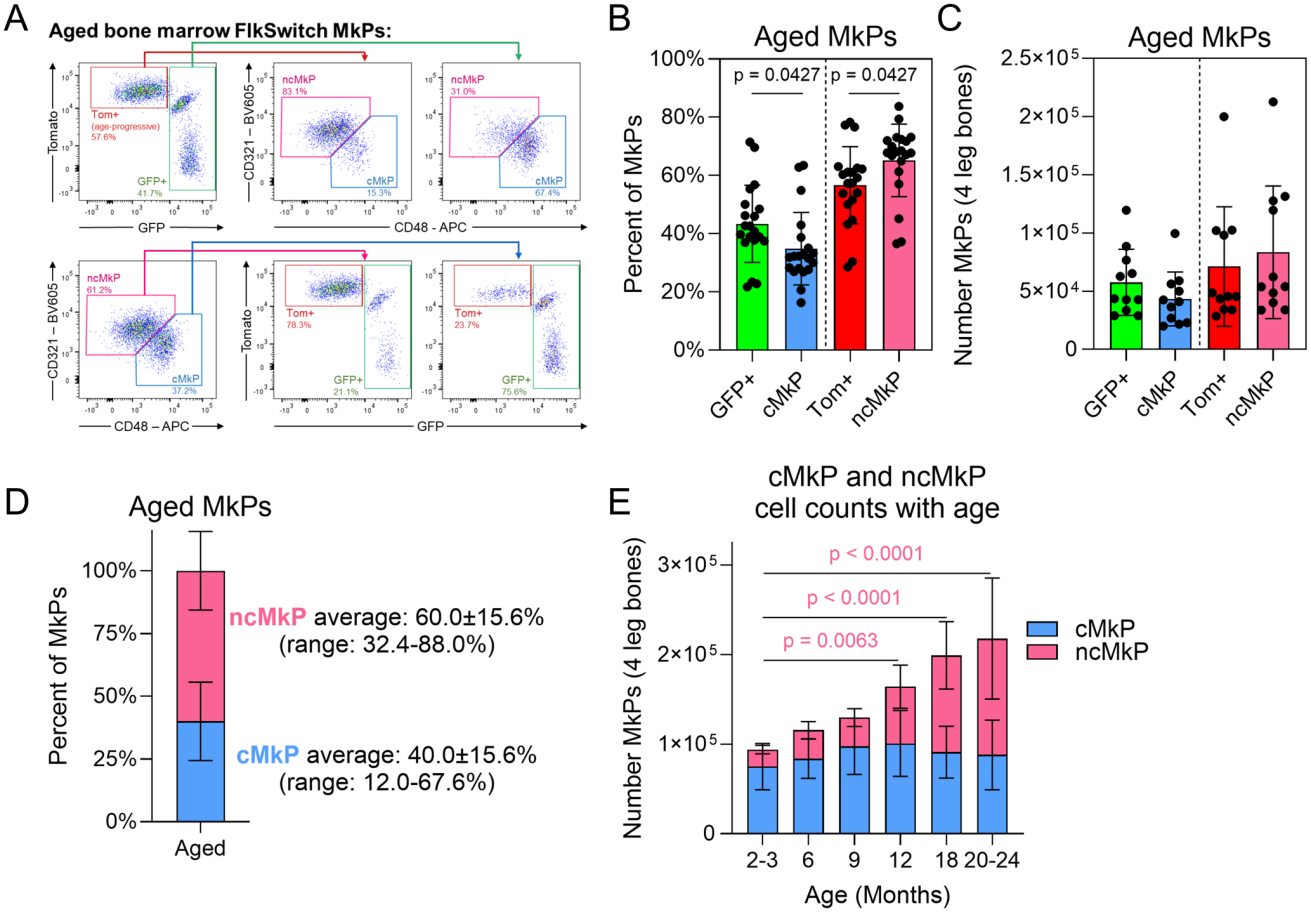
CD48 and CD321 efficiently enrich for GFP+ and Tom+ MkPs in old mice. (A) Example flow cytometry plots demonstrating the efficiency of using CD48 and CD321 in combination to enrich for aged GFP+ and Tom+ MkPs among FlkSwitch mice. The (B) frequency and (C) number of aged MkPs defined as GFP+, Tom+, cMkP, or ncMkP. Unpaired *t*‐tests comparing GFP+ to cMkP and Tom+ to ncMkP. (B) *n* = 20 across 12 independent experiments and (C) *n* = 11 across five independent experiments. (D) Frequency of cMkP and ncMkP among aged MkPs from wild‐type (WT) mice. *n* = 43 across more than 10 independent experiments. (E) Number of cMkPs and ncMkPs among WT mice at the ages indicated. *n* = 11, 12, 12, 17, 12, and 14 for ages 2 to 3, 6, 9, 12, 18, and 20 to 24 months, respectively, across 10 independent experiments. cMkPs and ncMkPs were compared individually by one‐way ANOVA with Dunnett's multiple comparisons test comparing all populations against the 2 to 3 month group. Data is stacked for visual simplicity. Color of *p* values indicates statistically significant comparisons.

To assess the combined ability of CD48 and CD321 to enrich for GFP+ and Tom+ MkPs in aged FlkSwitch mice, we first evaluated aged FlkSwitch BM (Figure 2A, top row). Remarkably, GFP+ MkPs were largely CD48+ CD321‐ whereas Tom+ MkPs exhibited the inverse CD48‐CD321+ expression pattern. Of note, cells positive for both Tom and GFP have undergone Flk2‐mediated Cre‐recombinase activity (allowing GFP expression) but have not yet fully degraded the Tom protein. Paired analyses indicate that Tom+ GFP+ MkPs statistically differ from Tom‐GFP+ MkPs with respect to CD48 and CD321 distribution, although the magnitude of difference is relatively minor and likely relates to the rate at which Tom protein is degraded in relation to the lifespan of individual MkPs (Figure S2A,B). Importantly, the enrichment for CD48+CD321‐ MkPs is maintained among GFP+ MkPs regardless of lingering Tom expression. Thus, we include Tom+GFP+ cells in the GFP+ gate as they have genetically and permanently “switched” from Tom to GFP expressing cells (Boyer et al. 2011) (Figure S1A).

We then performed the reverse approach, first separating aged FlkSwitch MkPs into CD48+CD321‐ and CD48‐CD321+ subpopulations and assessing the GFP+ and Tom+ staining profiles (Figure 2A, bottom row). Reassuringly, CD48+CD321‐ MkPs were largely GFP+ and CD48‐CD321+ MkPs were primarily Tom+. Thus, CD48 and CD321 selectively enrich for GFP+ and Tom+ MkPs among aged FlkSwitch mice. Importantly, the degree of separation conferred by CD48 and CD321 allows for prospective isolation via flow cytometry, enabling downstream applications. Given that GFP+ MkPs are generated via the canonical differentiation pathway, and Tom+ MkPs via an alternate, age‐progressive noncanonical pathway (Figure 1A), we term CD48+CD321‐ (GFP‐like) MkPs as canonical MkPs (cMkPs) and CD48‐CD321+ (Tom‐like) MkPs as noncanonical MkPs (ncMkPs).

The exceptional enrichment of aged GFP+ and Tom+ MkP populations by CD48 and CD321 is remarkably efficient as the overall frequencies of each related population are similar, although statistically different (comparing GFP+ to cMkPs and Tom+ to ncMkPs at the population level, Figure 2B). Importantly, this difference is minimal as cellular quantification demonstrates no statistically significant differences between the number of aged GFP+ MkPs and cMkPs or Tom+ MkPs and ncMkPs (Figure 2C). A minor fraction of cells does not conform to this alternative identification scheme. Indeed, when assessed intramouse, the frequency and number of each subpopulation within individual mice reveal minor, yet statistically significant, differences (Figure S2C,D). Overall, CD48 and CD321 possess the stark potential to significantly enrich for aged GFP+ and Tom+ MkPs.

We next sought to evaluate if our cMkP and ncMkP approach recapitulated our previous findings of aged FlkSwitch MkPs. First, we assessed the frequency of aged cMkPs and ncMkPs, which were similar to our previous report (Poscablo et al. 2024) (Figure 2D). The number of ncMkPs increased with advancing age and was statistically significant at 12 months of age (Figure 2E), consistent with the progressive increase in Tom + MkPs and platelets with age (Poscablo et al. 2024). Importantly, similar to GFP+ MkPs, the number of cMkPs did not change with age. Thus, cMkPs and ncMkPs faithfully recapitulate features of the aged MkP compartment previously only measurable with the FlkSwitch mouse model.

### 2.3 Expression of CD48 Protein, but Not RNA, Enriches for Aged GFP+ and Tom + MkPs Transcriptomically

**FIGURE 3:**
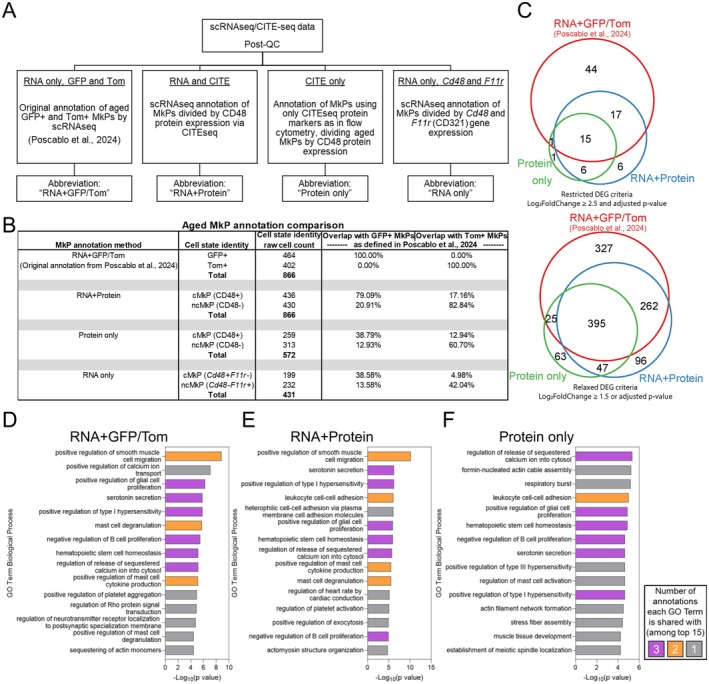
Aged cMkP and ncMkP transcriptomic signatures recapitulate their analogous GFP+ and Tom+ counterparts. (A) Schematic of additional transcriptomic MkP subtype annotations, including abbreviations used throughout the text and figures. (B) Comparison of the four annotation methods and how they perform compared to the original aged GFP+ and Tom + MkPs identified via “RNA + GFP/Tom” annotation (Poscablo et al. 2024). (C) Venn diagrams comparing the overlap in the numbers of DEGs generated by the three best annotation methods, comparing either aged GFP+ and Tom+ or cMkP and ncMkP populations. Top, DEG comparisons using strict statistical thresholds. Bottom, DEG comparisons using relaxed statistical thresholds. (D–F) GO Term Analysis between aged MkP subpopulations across three annotation methods. Top 15 GO Terms displayed. (D) GFP+ and Tom+ MkPs from the “RNA + GFP/Tom” annotation. (E) cMkPs and ncMkPs from the “RNA + Protein” annotation. (F) cMkPs and ncMkPs from the “Protein only” annotation.

To assess the concordance of cMkP and ncMkP enrichment of aged GFP+ and Tom+ MkPs with respect to transcriptional identity, we generated three additional annotations of our scRNA/CITEseq dataset parallel to the original “RNA + GFP/Tom” annotation (Poscablo et al. 2024) (Figure 3A). First, we subdivided all scRNAseq‐annotated MkPs by CD48 protein expression; CD321 was not available for CITEseq (“RNA + Protein,” Figure S3A). Second, we annotated MkPs using only CITEseq (protein) markers, similar to flow cytometry, and subdivided MkPs based on CD48 protein expression (“Protein only,” Figure S3B). As the “Protein only” annotation is the only one that does not rely on RNA‐based MkP identification, we assessed if this method accurately captured the transcriptomic MkP cell state. Encouragingly, 93.7% of aged “Protein only” MkPs identified were also classified as MkPs by scRNAseq. Third, using the original “RNA + GFP/Tom” annotation, we instead divided all scRNAseq‐annotated MkPs by RNA expression of *Cd48* and *F11r* (CD321): *Cd48* +*F11r*‐ cMkPs and *Cd48*‐*F11r* + ncMkPs (“RNA only” Figure S3C).

Each new annotation identified aged cMkPs and ncMkPs, thus we next sought to compare their transcriptomic fidelity. To do so, we compared each annotation method against the original scRNAseq‐based “RNA + GFP/Tom” annotation that identifies aged Tom+ MkPs from GFP+ MkPs (Poscablo et al. 2024). As each cell in scRNA/CITEseq data is individually barcoded, we compared how often a cell is captured in the “correct” bin across MkP annotations (Figure 3B). That is, of all aged scRNAseq‐annotated GFP+ and Tom+ MkPs, how many were also defined as cMkPs and ncMkPs, respectively?

We found that the “RNA + Protein” annotation method performed best compared to the “RNA + GFP/Tom”. The “Protein only” annotation largely correctly captures GFP+ and Tom+ cells, yet is more conservative in total numbers of cells identified compared to the “RNA + GFP/Tom” annotation. The “RNA only” annotation performed poorest, capturing less than 50% of GFP+ or Tom+ MkPs in either of the cMkP or ncMkP bins and was removed from further assessment. This may indicate that CD48 (and CD321) protein profiles of aged cMkPs and ncMkPs identify those cell states better than their corresponding RNA profiles, a significant advantage due to the ability to prospectively and simply isolate transcriptomically unique MkPs. Next, we generated restrictive and relaxed DEG lists between aged cMkPs and ncMkPs from the “RNA+ Protein” and “Protein only” annotations to assess the amount of overlap with the “RNA + GFP/Tom” annotation (Figure 3C, Table S3). Remarkably, for both restricted and relaxed statistical cutoffs, we find high levels of concordance, indicating that the cMkP and ncMkP cell definitions largely recapitulate aged GFP+ and Tom+ MkPs transcriptomically.

We then subjected the DEG lists from each annotation to GO Term analysis (Figure 3D–F). Given that the small number of DEGs present in restricted DEG lists does not constitute enough statistical power, we utilized the DEGs generated by our relaxed statistical criteria. Importantly, the GO Terms for each annotation returned several overlapping categories, including those related to HSC homeostasis, proliferation, and activation, further supporting the robustness of cMkP and ncMkP designations to recapitulate GFP+ and Tom+ MkPs transcriptomically. Thus, the cMkP and ncMkP paradigm of aged MkPs robustly recapitulates that of their GFP+ and Tom+ counterparts transcriptomically.

### 2.4 Young Mice Possess Rare ncMkPs

As part of testing CD48 and CD321 to isolate aged GFP+ and Tom+ MkPs, we performed parallel analyses with young (2–3 month) mice. The majority of MkPs in young BM are classically derived via transition through a Flk2‐expressing progenitor cell stage and demonstrate high levels of Cre recombinase‐mediated floxing and resulting GFP expression in FlkSwitch mice (Poscablo et al. 2024; Boyer et al. 2011) (Figure 1A), meaning that young MkPs are primarily GFP+. When we assessed cMkPs and ncMkPs in young mice, we detected a rare, yet consistent, population of ncMkPs, similar to what others have demonstrated (Carrelha et al. 2024; Li et al. 2024; Morcos et al. 2022) (Figure 2E 2‐3 month age group and Figure S4A). Young ncMkPs constitute ~20% of total MkPs, a significantly smaller frequency than in old mice (Figure S4B). The absolute cellular rarity of young ncMkPs precludes our ability to assess them robustly in our scRNA/CITEseq data, yet similar populations of young CD48+ and CD48‐ MkPs were assessed by Morcos et al. (2022) and Carrelha et al. (2024).

Carrelha et al. (2024) also observed young MkP phenotypic heterogeneity following single HSC transplantations, including CD48 and CD24a, each of which enriches for predicted canonical (CD48) or platelet‐biased (CD24a) differentiation trajectories. Therefore, we tested if CD48/CD24a enriches for MkP subpopulations similar to CD48/CD321 in young and aged mice (Figure S4C,D). Indeed, CD321 and CD24a are interchangeable (Figure S4E,F). To ensure each identification method enriched for the same cells, we further evaluated the ability of each combination to appropriately represent Tom+ and GFP+ MkPs from FlkSwitch mice (Figure S4G–J). Only among rare Tom+ MkPs in young mice did we observe statistically significant differences between CD321 and CD24a, yet the magnitude of difference is negligible. Thus, heterogeneity among MkPs is present in young adult mice, CD321 and CD24a can be used interchangeably, and the pool of ncMkPs specifically expands upon aging (Figure 2E).

### 2.5 Female Mice Exhibit Modestly Reduced Effects of Aging to the Megakaryocytic Lineage

The highest efficiency of Cre‐recombinase activity is found only in male FlkSwitch mice, thus we have omitted female FlkSwitch mice from analyses that rely on detection of GFP and Tom (Poscablo et al. 2024). In contrast, this new cMkP and ncMkP paradigm allows for high fidelity proxy detection and thus we assessed if there are sex dimorphisms among MkP abundance throughout life. Total MkP count and BM frequency in male versus female wild‐type (WT) mice were equivalent across six age groups (Figure S5A). Male mice alone had a detectable MkP expansion by 12 months of age by cell count and 9 months of age by frequency, whereas comparing only female mice demonstrated a lag, only becoming statistically significant around 20 months of age with respect to cell numbers yet detectable by expanded frequency at 12 months (Figure S5B,C). There were no robust differences between male and female mice for cMkPs or ncMkPs (Figure S5D,G). Both male and female mice individually maintained equivalent cMkP numbers and frequency throughout life (Figure S5E,F) and demonstrated age‐related ncMkP expansions with statistically detectable increases occurring by 12 months of age in male mice (cell count and frequency) and 18 and 12 months for count and frequency in females, respectively (Figure S5H,I). Overall, no major differences in the number or relative abundance of MkPs were found between male and female mice.

Murine platelet counts increase with age via the unique age‐progressive pathway (Poscablo et al. 2024). Thus, we also examined total platelet count among male and female WT mice, hypothesizing they would be elevated with aging in both, reflecting the age‐related ncMkP expansion. Indeed, platelet counts were elevated upon aging (Figures S5J and S11G). Interestingly, female mice maintained consistently lower platelet counts compared to their male counterparts (Figure S5K). Although age‐related expansion of platelet numbers was detected in both sexes, female mice exhibit expansion to a reduced degree (Figure S5L,M). Thus, the increase in BM MkPs upon aging results in increased peripheral blood platelets.

### 2.6 ncMkPs Uniquely Gain Survival, but Not Proliferative, Advantage With Age

**FIGURE 4:**
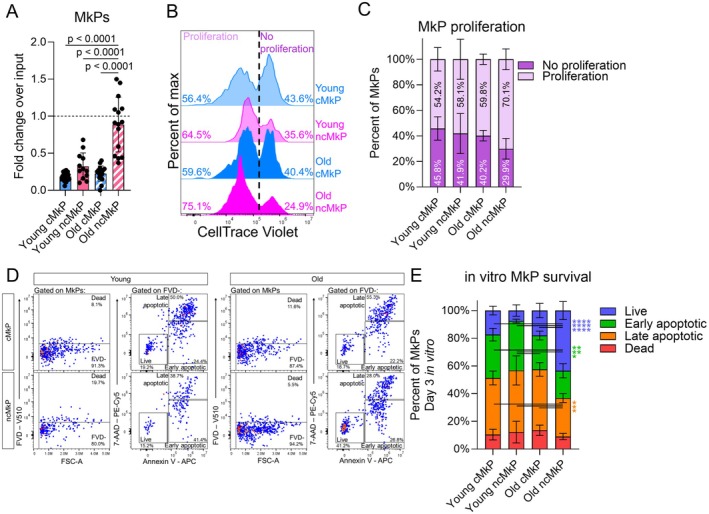
ncMkPs gain a survival advantage with age. (A) Fold change over input (2000 MkPs) for the number of phenotypic MkPs recovered following 3 days in culture. Each point represents the average of up to three technical replicates as cell number allowed. *n* = 12 to 17 mice across nine independent experiments. One‐way ANOVA adjusted for multiple comparisons via Tukey's test. (B) Histogram representations of CellTrace Violet (CTV) distribution among MkPs cultured in vitro for 3 days. (C) Proportion of in vitro cultured MkPs that proliferated or not over the 3 day culture. *n* = 3 to 4 across four independent experiments. Up to three technical replicates per mouse were averaged together. Statistical nonsignificance was determined via one‐way ANOVA adjusted for multiple comparisons via Tukey's test, no proliferation and proliferation groups tested separately. Data is stacked for visual simplicity. Percents indicate mean values for each proliferative status for each age and cell type group. (D) Representative flow cytometry plots of in vitro viability determination following 3 days of culture. Cells were defined as dead if they were fixable viability dye (FVD) positive. Determination of nondead cell states was made among cells that were FVD negative. (E) Proportion of viability cell states of 3 day cultured MkPs. *n* = 3 to 4 across four independent experiments. Up to three technical replicates per mouse were averaged together. *p < 0.05, **p < 0.01, and ****p < 0.0001 by one‐way ANOVA adjusted for multiple comparisons via Tukey's test, each cell state tested separately with lines indicating comparisons within each cell state. Data is stacked for visual simplicity.

Given the rare, yet stable, presence of ncMkPs in young mice, we next sought to uncover if MkP subpopulations in both young and old mice were functionally distinct. We first performed in vitro recovery analyses as we previously demonstrated that aged Tom+ MkPs have superior performance (Poscablo et al. 2024). We sorted cMkPs and ncMkPs from young and old WT mice and, following 3 days of culture, determined the number of viable MkPs remaining. Similar to old Tom+ MkPs, old ncMkPs significantly outperformed all other MkP subsets in recovered viable cell counts (Figure 4A and Figure S11K). The significant in vitro recovery of aged ncMkPs compared to all other MkP subsets was similar between male and female mice, with only slight differences between young cMkPs (Figure S6A,B). Of note, young ncMkPs do not have a functional advantage in vitro, highlighting the specific effect of aging on this compartment.

We then sought to uncover the mechanism(s) underlying the enhanced in vitro performance of aged ncMkPs. Proliferation and homeostasis (including survival) were among the top hits revealed by GO Term analyses (Figure 3D–F). Thus, we hypothesized that aged ncMkPs have a proliferative and/or survival advantage. We assessed in vitro proliferation with CellTrace Violet (CTV), a fluorescent dye that, upon each cell division, diminishes in intensity via dilution. Sorting and culturing CTV^high^ MkPs (Figure S11J) revealed no significant differences in the frequency of cells undergoing at least one cell division across all four MkP subpopulations (Figure 4B,C). We further evaluated the MFI of CTV only among cells that proliferated as a surrogate for the median number of proliferative events (more dye dilution/reduced MFI equates to a greater number of times a given population proliferated). We found statistically significant, yet low magnitude shifts indicating that young cMkPs and old ncMkPs may be equivalently slightly more proliferative than the other populations (Figure S6C). Therefore, an increased number of cell divisions could not explain the higher numbers of recovered ncMkPs upon in vitro culture.

Next, we evaluated in vitro MkP survival via combinatorial viability assessment (Figure 4D). This revealed that aged ncMkPs specifically possess a significant survival advantage compared to all other MkPs, which were equivalent to each other (Figure 4E). Viability evaluation was performed simultaneously with CTV, allowing us to determine if MkP viability cell state influences proliferative potential. We stratified the proliferation data across the live, early apoptotic, and late apoptotic cell states (Figure S6D–I). Interestingly, nearly all live MkPs proliferated, whereas those destined for apoptosis demonstrate a range of proliferative activity (Figure S6D–F) and magnitude (Figure S6G–I). Of note, young ncMkPs, particularly those not undergoing apoptosis, appear to be far less proliferative than any other subset (Figure S6C,G). Thus, MkP subset proliferation and survival functions are likely uncoupled (Figure S6J).

### 2.7 ncMkPs Are Functionally Distinct From cMkPs In Vivo and Demonstrate Enhanced Function During Aging

**FIGURE 5:**
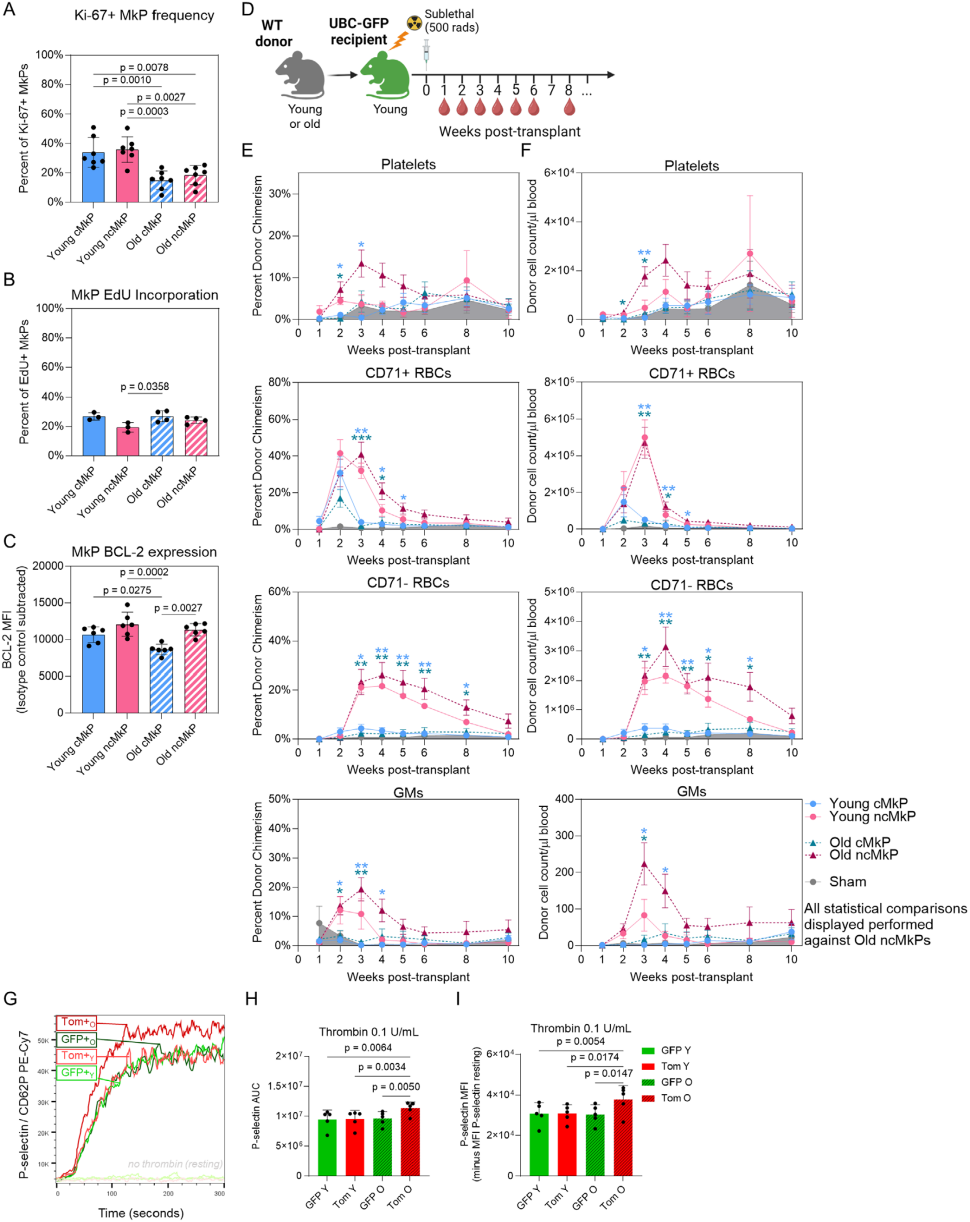
ncMkPs have distinct functional capacity from cMkPs and gain enhanced platelet specification ability upon aging. (A–C) Flow cytometry analysis of MkP populations from freshly isolated BM. Each point represents an individual mouse, and the frequency or MFI is background‐subtracted from an isotype or negative control. One‐way ANOVA adjusted for multiple comparisons via Tukey's test. (A) Frequency of cells expressing Ki‐67. *n* = 7 across five independent experiments. (B) Frequency of cells incorporating EdU 24 h postinjection. *n* = 3 to 4 across four independent experiments. (C) Relative BCL‐2 protein abundance as determined by standardized flow cytometry (Manso and Medina 2021). *n* = 6 across five independent experiments. (D) MkP transplantation experimental design. The (E) percent donor chimerism and (F) donor‐derived cell number/μl of blood for the indicated populations. Shaded area represents the sham control. Each point represents the mean ± standard error of the mean (SEM). See Figure S8A–D, five independent experiments. *p < 0.05, **p < 0.01, and ***p < 0.001 by two‐way ANOVA adjusted via Dunnett's multiple comparisons test, comparing each group at each time point to the aged ncMkPs for display simplification. (G) Representative P‐selectin neo‐exposure kinetic plot for resting and activated washed platelets in whole blood from young and old FlkSwitch mice. Platelets from young and old mice were activated together with 0.1 U/mL human thrombin. P‐selectin neo‐exposure over time by (H) the area under the curve (AUC) and (I) MFI of activated young (*n* = 4) and old (*n* = 5) FlkSwitch mice by thrombin (0.1 U/mL). Statistical significance determined by one‐way ANOVA with Tukey's multiple comparisons test.

In vitro regulation of MkPs may differ from in vivo. To assess in vivo regulatory mechanisms of young and old cMkPs and ncMkPs, we first sought to evaluate in vivo proliferation and survival given both the GO Term Analyses and in vitro findings (Figures 3D–F and 4). Analysis of Ki‐67, a marker preceding entry into S phase of the cell cycle, from freshly isolated BM revealed a higher frequency of Ki‐67+ cells among young MkP subsets compared to old (Figure 5A and Figure S11C). This potentially indicates that young MkPs proliferate more than aged, or that aged MkPs progress faster through these cycles, thus downregulating Ki‐67 more quickly, which would not be captured in this “snapshot” analysis. We do not suspect that this finding is due to endomitosis, as MkPs are diploid (Nakorn et al. 2003). Indeed, when assessed, young and old cMkPs and ncMkPs contained less than 1% of cells that were 8n+ (Figures S7A and S11F). When sex differences were assessed, slightly more young cMkPs from female mice were found to be Ki‐67+ than their male counterparts (Figure S7B,C). Additionally, paired analysis between cMkPs and ncMkPs from the same mice revealed no Ki‐67 differences between young cMkP and ncMkP populations and a slight, but significant, increase in old ncMkPs compared to cMkPs (Figure S7D).

Cell cycle inference based on hallmark scRNAseq signatures indicates minor differences between young and old cMkP populations, with aged ncMkPs possessing fewer cells in a proliferative state (Figure S7E). To gain a better understanding of the in vivo proliferative nature of MkPs, we performed 24 h EdU pulse‐chase experiments in young and old WT mice, aiming to measure how often MkPs proliferate during this time (Figure 5B and Figure S11D). Overall, young and old cMkPs and ncMkPs incorporated EdU equivalently, with the exception of slightly diminished incorporation in young ncMkPs, as additionally revealed by paired analysis within individual mice (Figure S7F). Importantly, this finding of diminished proliferative capacity specifically among young ncMkPs aligns with our in vitro analysis of young ncMkPs (Figure S6C,D,G). Thus, in vitro and in vivo analyses of young and aged cMkPs and ncMkPs did not reveal major differences in steady‐state proliferation, with the exception of young ncMkPs that may be more quiescent.

Aged ncMkPs demonstrated a survival advantage in vitro (Figure 4D,E). Analysis of the bulk and scRNAseq data indicated that gene expression of *Bcl2*, which codes for a major antiapoptotic protein, was significantly higher in aged Tom+/ncMkPs compared to young and aged GFP+/cMkPs (Figure S7G,H). Analysis of freshly isolated MkPs via standardized flow cytometry (Manso and Medina 2021) revealed high levels of BCL‐2 protein among all MkP subpopulations, with old cMkPs possessing slightly, but significantly, reduced levels compared to other MkP subpopulations (Figure 5C and Figure S11E). Paired analysis demonstrated slightly higher BCL‐2 protein levels in ncMkPs compared to cMkPs within each age group (Figure S7I). Bulk and single‐cell transcriptomic evaluation of other apoptotic and stress response pathways did not reveal any robust differences (Figure S7J). We also assessed MkP viability from freshly isolated BM (Figure S7K). Unsurprisingly, the vast majority of cells were highly viable given that dead cells are cleared rapidly in vivo and the likely in situ inhibition of apoptosis by BCL‐2. Thus, although difficult to measure in vivo, key molecular features of proliferation and survival among young and aged cMkPs and ncMkPs are grossly similar.

MkPs primarily give rise to megakaryocytes and subsequently platelets in vivo. Given that age‐progressive Tom+ MkPs have enhanced platelet‐producing ability following transplantation (Poscablo et al. 2024), we next tested if aged ncMkPs have similarly elevated in vivo performance. We performed transplantations of cMkPs and ncMkPs from young and aged mice into sublethally irradiated young recipients and longitudinally assessed blood cell output (Figure 5D–F and Figures S8A–D and S11G,H). We expected aged ncMkPs (analogous to aged Tom+ MkPs) to outperform all other populations with respect to platelet generation, subsequently comparing all other conditions to aged ncMkPs. Indeed, aged ncMkPs transiently generated more platelets than young or old cMkPs by measures of donor chimerism and total number of platelets/μl blood. Similar to our in vitro findings, young ncMkPs were indistinguishable from cMkPs (young and old). Thus, aged ncMkPs uniquely possess enhanced functional capacity with respect to platelet output following transplantation, recapitulating the output of the analogous age‐progressive Tom + MkPs (Poscablo et al. 2024).

We previously demonstrated that, upon transplantation, MkPs can transiently give rise to erythroid and/or granulocyte/monocyte (GM) lineages (Poscablo et al. 2024, 2021). Assessment of CD71+ and CD71‐ erythrocytes revealed that the red blood cell (RBC) output is primarily driven by ncMkPs, regardless of age (Figure 5E,F), recapitulating MkP transplants performed by Morcos et al. 2022, who also demonstrated that young CD48‐ MkPs possess an erythroid gene signature in addition to the expected megakaryocyte‐associated genes. Similarly, GM output following MkP transplantation primarily arises from both young and old ncMkPs, again similar to Morcos et al. (2022). As expected, no B or T cell readout was detected (Figure S8C,D). Thus, ncMkPs from young and old mice are functionally distinct from their cMkP counterparts in the context of transplantation and, upon aging, gain additional capacity for platelet generation.

We next wanted to assess if platelets arising from young ncMkPs were hyperactive, as we have previously shown that Tom+ platelets from aged FlkSwitch mice uniquely demonstrated hyperreactivity compared to young and old GFP+ platelets (Poscablo et al. 2024). We are currently unable to identify cMkP‐ or ncMkP‐derived platelets in WT mice, so we assessed the functionality of young and old Tom+ and GFP+ platelets from FlkSwitch mice. Young FlkSwitch mice possess a rare, yet statistically indistinguishable population of Tom+ platelets in peripheral blood (Poscablo et al. 2024; Carrelha et al. 2024) that may represent the earliest stages of the age‐progressive pathway and those that escaped Cre‐mediated floxing. To that point, the cMkP/ncMkP phenotypic CD48/CD321 paradigm largely applies to young MkPs as well (Figure S8E–G). Although population level and intra‐mouse assessment revealed statistical differences between GFP+/cMkP and Tom+/ncMkP populations, the magnitude of their differences is marginal. Thus, young Tom+ platelet function likely represents those that are primarily ncMkP‐derived. Following ex vivo stimulation with either thrombin (Figure 5G–I) or ADP (Figure S8H,I), we found that aged Tom+ platelets are uniquely hyperreactive, while young Tom+ platelets perform equivalently to those of young and aged GFP+ platelets. Thus, ncMkPs uniquely produce more platelets with enhanced reactivity only upon aging.

### 2.8 Aged, but Not Young, HSCs Efficiently Produce ncMkPs

Young MkPs are primarily specified via the classical path of hematopoiesis that requires transition through a Flk2‐expressing cell state typically associated with loss of *bona fide* HSC identity (Manso et al. 2024; Poscablo et al. 2024; Christensen and Weissman 2001) (Figure 1A). Rare, yet functionally indistinguishable, ncMkPs may also directly arise from HSCs in young mice (Carrelha et al. 2024; Li et al. 2024; Morcos et al. 2022) (Figure S8E–G). However, upon aging, the distinct HSC‐to‐MkP direct pathway dramatically expands with functionally enhanced MkPs (Figure 1A) (Poscablo et al. 2024). We therefore sought to uncover if young and old HSCs differentially generate cMkPs and ncMkPs, if removing the aged environment influences specification, and whether HSCs are clonally restricted to generate either MkP subtype.

**FIGURE 6:**
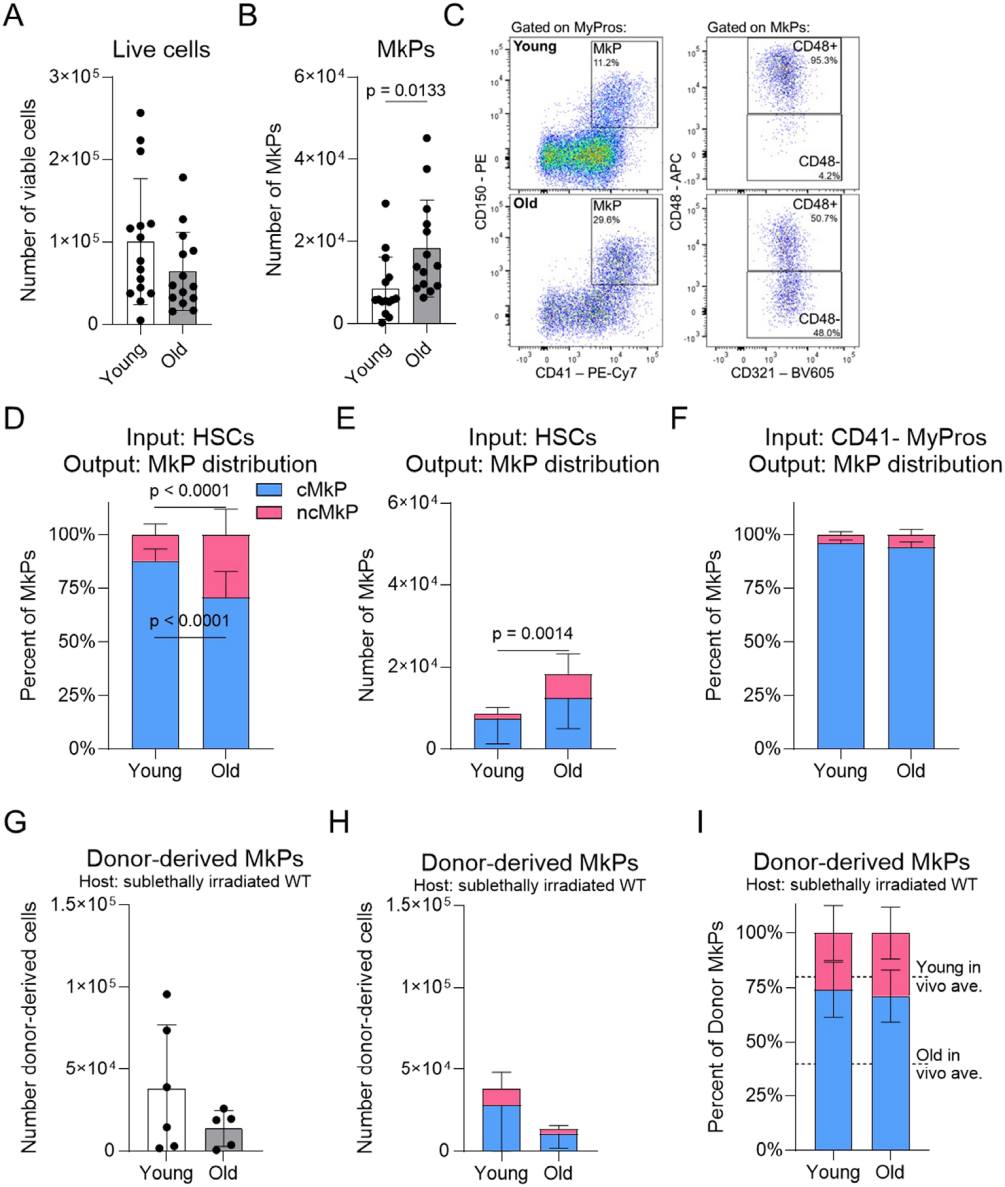
Aged HSCs specifically generate ncMkPs and restore output balance upon exposure to a young environment. (A, B) Number of (A) total live cells or (B) phenotypic MkPs generated following 7 days of in vitro HSC culture. Each point represents the average of three technical replicates from individual mice. *p < 0.05 by unpaired *t*‐test. *n* = 15 across nine individual experiments. (C) Representative flow cytometry plots of in vitro HSC‐derived phenotypic MkPs, cMkPs, and ncMkPs. (D, E) Proportion (D) and number (E) of cMkPs and ncMkPs generated by HSCs following 7 days of in vitro culture. n and number of experiments as in (A, B). Unpaired *t*‐test; data is stacked for visual simplicity. (F) Frequency of cMkPs and ncMkPs generated by CD41‐ MyPros following 7 days of in vitro culture. Statistical nonsignificance by unpaired *t*‐test. *n* = 5 across three individual experiments. Data is stacked for visual simplicity. (G–I) Young or old HSCs were transplanted into young sublethally irradiated (500 Rad) young WT hosts. After 16 to 20 weeks, BM was analyzed for the (G) number of donor‐derived total MkPs and (H, I) number and frequency of cMkPs and ncMkPs, respectively. Each point represents an individual mouse. *n* = 6 young and 5 old from two independent experiments. Statistical nonsignificance by unpaired *t*‐test. Data is stacked for visual simplicity.

We first established an in vitro cell culture assay that is permissive (but not restrictive) to megakaryocytic lineage differentiation, effectively normalizing the extrinsic environment. HSCs (Lin^low^cKit+Sca1+CD150^high^Flk2[CD135]‐, Figure S11A,J) were sorted from young and old WT mice and cultured for 7 days. At the end of the culture period, the total number of live cells produced was equivalent from young and old HSCs (Figure 6A and Figure S11J), yet numbers of phenotypic MkPs were elevated over two‐fold in aged HSC cultures, analogous to the in vivo state (Figure 6B,C, left panels). We also detected phenotypic platelets (small, Lineage‐CD150+CD41+, Figures S9A and S11L); such in vitro‐derived entities have been shown to be phenotypically and functionally similar to freshly isolated platelets (Nishikii et al. 2008; Ungerer et al. 2004). We then asked if we could identify cMkPs and ncMkPs in vitro using CD48 and CD321. Among phenotypic MkPs, all cells had equivalent CD321 expression (Figure 6C, right panels). In contrast, expression of CD48 recapitulated the pattern observed in vivo by us and others (Carrelha et al. 2024; Morcos et al. 2022): MkPs generated from young HSCs were primarily CD48+ (mean 87.9%, range 75.5%–96.7%) whereas MkPs produced by aged HSCs contained distinct populations of CD48+ (mean 70.6%, range 50%–91.5%) and CD48‐ (mean 29.4%, range 8.3%–50.0%) MkPs (Figure 6C,D). Indeed, the distribution of in vitro, HSC‐derived CD48+ cMkPs and CD48‐ ncMkPs recapitulated our in vivo observation of an age‐dependent increase in ncMkPs (Figure 6D, compare to Figure S4B). Importantly, young and old HSCs generated equivalent numbers of cMkPs, yet aged HSCs produced significantly more ncMkPs, recapitulating our in situ observations (Figure 6E).

Canonical, but not HSC‐direct, MkP differentiation progresses from HSCs through intermediate progenitor cell states, including myeloid progenitors (MyPros), a heterogeneous collection of myeloid primed cell states (Figure 1A) (Manso et al. 2024). To test if myeloid progenitors are capable of producing phenotypic ncMkPs in vitro, we sorted CD41‐ MyPros (to omit MkPs) and subjected them to the same in vitro assay as HSCs (Figure 6F). Strikingly, both young and old MyPros almost exclusively generated cMkPs, with no difference in distribution.

To expand upon our in vitro assays that removed the aged environment as a potential factor influencing MkP subtype specification and test if exposing aged HSCs to a young environment in vivo affects ncMkP generation, we performed HSC transplantations from young and old mice into sublethally irradiated young recipients (Figure 6G–I and Figure S11I). Although the total numbers of donor‐derived MkPs at 16 to 20 weeks posttransplantation tended to be reduced in old recipients, as expected, neither the total numbers of MkP (Figure 6G) nor the cMkP and ncMkP numbers (Figure 6H) were significantly different. Importantly, both young and aged HSCs produced indistinguishable cMkP:ncMkP ratios, and those ratios reflect young steady‐state BM (Figure 6I, compare to Figure S4B). Collectively, exposure of old HSCs to a young in vivo environment appears to overcome the intrinsic capacity for ncMkP production that is evident in situ and in vitro.

**FIGURE 7:**
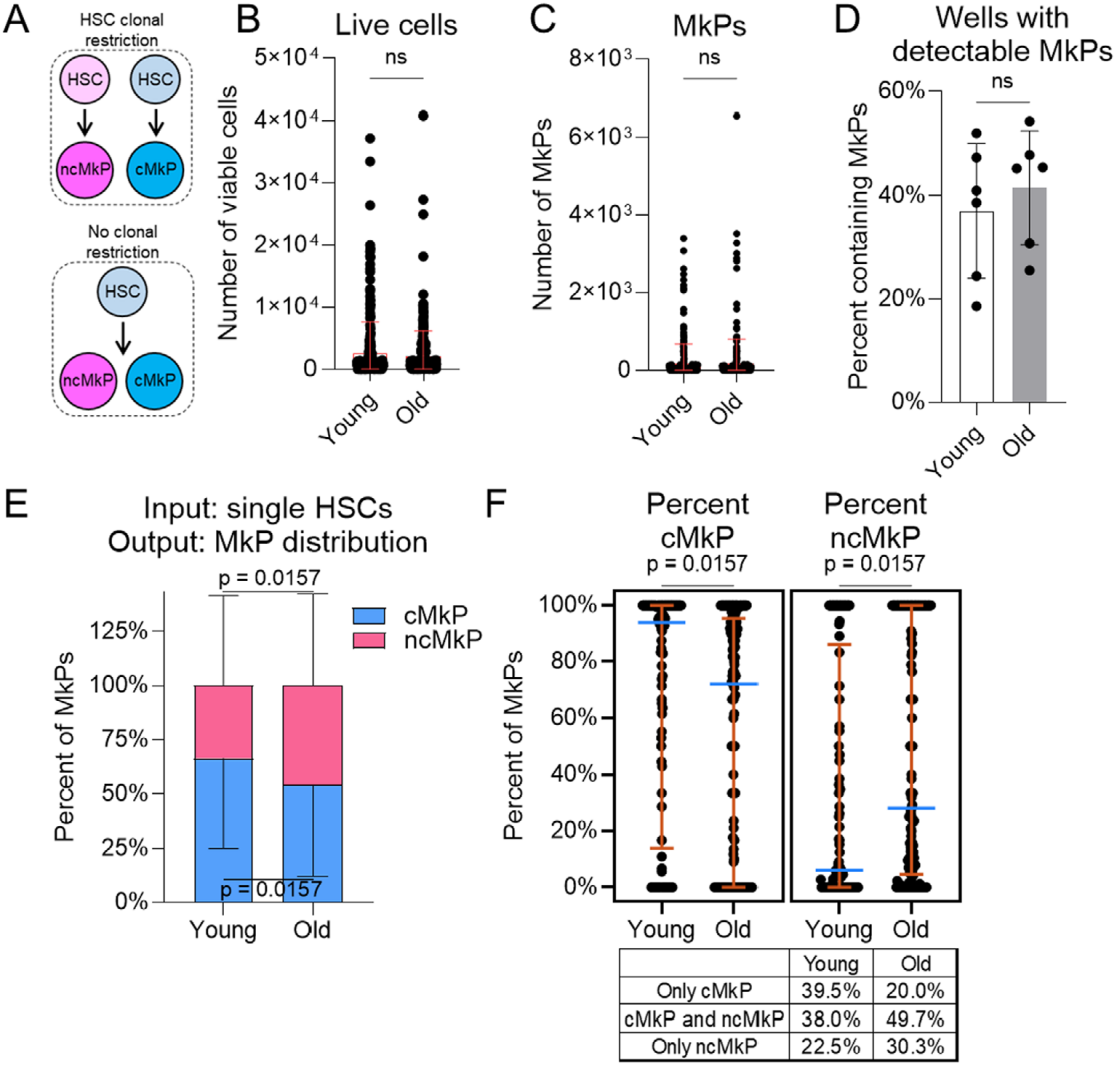
ncMkPs are not exclusively generated by clonally restricted HSCs. (A) Model of potential HSC clonal restriction to MkP subtype. (B–F) Definition of HSCs assessed is Lin^low^cKit+Sca1+CD150^high^Flk2‐CD48‐. Number of (B) total live cells or (C) phenotypic MkPs generated following 7 days of in vitro single cell HSC culture. Each point represents an individual input HSC. Statistical nonsignificance by unpaired *t*‐test. Young *n* = 256 and old *n* = 270 individual HSCs from five individual mice across three individual experiments. (D) Among single HSC cultures, the frequency of HSCs giving rise to at least one phenotypic MkP. Each point represents an individual mouse, *n* = 6 per age across four individual experiments. Statistical nonsignificance by unpaired *t*‐test. (E, F) MkP subtype distribution produced by single HSC cultures. (E) Overall MkP subtype distribution. Data is stacked for visual simplicity. (F) Granular analysis of data from (E) Frequency of cMkP (left) or ncMkP (right) among young and old single‐cultured HSCs. Blue line indicates the median, whereas the orange lines indicate quartiles. Each point represents an individually cultured HSC that gave rise to at least one phenotypic MkP. The table provides a summary of MkP output distribution from individual HSCs. *n* = 129 young and *n* = 155 old across six individual experiments. Statistical significance by unpaired *t*‐test.

We next sought to determine if young or aged HSCs are clonally restricted at the single‐cell level to generate cMkPs or ncMkPs (Figure 7A). Platelet lineage bias and restriction have been proposed for subsets of HSCs (Manso et al. 2024; Carrelha et al. 2024), and it remains possible that any predisposition is further constrained to specific MkP subpopulations that have different functional capacities. Using our established in vitro system (Figure 6A–F), we sorted individual HSCs (Lin^low^cKit+Sca1+CD150^high^Flk2‐CD48‐) from young and old WT mice into single wells of a 96‐well plate. Following 7 days in culture, the total number of live cells was similar (Figure 7B), mirroring the bulk experimental approach. Among all wells, the number of MkPs generated was variable, with no consistent differences detected between young or old individual HSCs (Figure 7C). Interestingly, this is in contrast to the bulk HSC cultures (compare to Figure 6B). The frequency of single HSCs that generated MkPs (~40%) was also similar between young and old cells (Figure 7D).

To assess if MkP subtype generation is clonally restricted to individual HSCs, we calculated the proportion of CD48+ and CD48‐ in vitro derived MkPs among wells with at least one detectable phenotypic MkP. If HSCs are clonally restricted, we would expect that all MkPs within a well would be exclusively either CD48+ or CD48‐ (Figure 7A). Conversely, if there was no restriction, we would expect variability in the ratio of CD48+:CD48‐ MkPs. Overall, single young HSCs generated a higher frequency of cMkPs (and less ncMkPs) than their aged counterparts (Figure 7E), mimicking the bulk data (compare to Figure 6D). A more granular analysis indicates that some young and aged single HSCs gave rise to both subsets, whereas others generated only one subtype (Figure 7F). The overall proportionality also indicates the expected age‐related shift to ncMkP generation by HSCs. Collectively, a strict clonal restriction model is not compatible with these results.

The phenotypic HSC definition we used (Lin^low^cKit+Sca1+CD150^high^Flk2‐) for bulk in vitro assays may contain rare MPP2 (Lin^low^cKit+Sca1+CD150^high^Flk2‐CD48+) cells that could contribute to MkP generation. We assessed the post‐isolation culture input cells and found that a small fraction was CD48+ (Figure S9B). However, although we only analyzed HSCs defined as CD48‐ in our single‐cell assays (Figure 7), our initial sort contained, but did not utilize, CD48. These indexed sorts therefore allow identification of the small percentage of identifiable wells that contain MPP2s (Figure S9C). When retroactive index‐based identification of wells initiated by MPP2s was identified and measured, we found that, compared to HSCs, MPP2s contribute minimally to in vitro MkP generation, do not differ in output or bias with age, and importantly, primarily generate cMkPs (Figure S9D–H, compare to Figure 7). Thus, the minimal presence of MPP2s in the bulk cultures is unlikely to significantly alter the outcome and may instead slightly mask the age‐dependent differences observed (Figure 6A–E).

Given that aged HSCs generate more ncMkPs at the bulk and single‐cell levels, we sought to predict if specific HSC phenotypes would give rise to cMkPs, ncMkPs, or both. Our single‐cell HSC sorting was indexed such that we know the relative expression level of each marker included in the sorting antibody cocktail and which well corresponds to each individual HSC. We then correlated the relative expression of key markers (cKit, CD150, Sca1, CD41, and CD321) with the number of viable cells, MkPs, and cMkP:ncMkP ratios (Figure S10, Table S4). Interestingly, no comparison revealed a consistent correlation. Thus, commonly used phenotypic marks among murine HSCs do not predict in vitro MkP subtype generation, which largely mirrors the in vivo state.

## 3 Discussion

Here, we uncovered new molecular, phenotypic, and functional aspects of age‐unique MkPs, including faithfully enriching for them without genetic reporters using CD48 and CD321 (Figure 2A). In doing so, we also discovered that phenotypic ncMkPs exist as a rare population in young mice that increase over time via the direct HSC “shortcut” path (Poscablo et al. 2024) (Figure 2E). Importantly, we have demonstrated for the first time that both male and female mice have similar megakaryopoiesis aging patterns (Figure S5). In contrast to functional decline often associated with aging, old ncMkPs gain enhanced survival (Figure 4E) and, upon transplantation, superior platelet producing capacity (Figure 5E,F). Aged ncMkPs, analogous to the age‐progressive Tom+ MkPs, appear to be directly produced by HSCs (Figure 6C–E), but not by MyPros which appear restricted to producing only cMkPs (Figure 6F). We also postulate that the age‐related rise of ncMkPs is not clonally restricted at the level of the HSC as some single HSC clones gave rise to both cMkPs and ncMkPs in vitro (Figure 7F). Thus, we have uncovered molecular and functional heterogeneity among young and aged MkPs, supporting and expanding upon previous reports (Poscablo et al. 2024; Carrelha et al. 2024; Li et al. 2024; Morcos et al. 2022). We conclude that aged CD48‐CD321+ ncMkPs recapitulate the age‐progressive, direct HSC‐derived Tom+ MkPs.

The cMkP/ncMkP paradigm of aged MkPs is a powerful method for investigating distinct pathways of megakaryopoiesis without the need for genetic lineage tracing mouse models. The addition of scCITEseq to our published bulk and scRNAseq of aged FlkSwitch BM HSPCs (Poscablo et al. 2024, 2021) revealed that stratification of aged MkPs by CD48 protein, but not RNA, best recapitulates the GFP+ and Tom+ subpopulations transcriptomically (Figure 3). Indeed, although a more conservative approach, protein‐only identification and separation of aged MkPs largely capture the canonical and age‐progressive MkPs previously only identifiable using FlkSwitch mice. Thus, cell sorting‐based experimental approaches utilizing CD48 and CD321 allow for faithful testing of aged GFP+ and Tom+ MkPs.

The idea of molecular and functional heterogeneity among hematopoietic stem and progenitor cell pools is largely accepted (Poscablo et al. 2024; Paul et al. 2016; Haas et al. 2018; Pietras et al. 2015). Our observation that young and old ncMkPs possess similar capacity for erythroid and myeloid cell output in a transplantation setting (Figure 5E,F) challenges the hypothesis that phenotypic MkPs (as currently defined) are unipotent platelet progenitors. However, we do not observe such production in the in situ aging state as old FlkSwitch mice do not possess Tom+ erythroid or myeloid cells (Poscablo et al. 2024). Thus, this additional functional capacity is only induced during transplantation and may reflect the ability of ncMkPs, but not cMkPs, to respond to the acute need for blood cell reconstitution following myeloablation and/or stress. It remains to be seen if perturbations shift ncMkP function to nonplatelet production in situ, but given that no other hematopoietic lineage cell is Tom+ during aging in FlkSwitch mice, we cannot conclude that phenotypic MkPs are “contaminated” with a CMP‐like cell. It is worth noting that cMkPs possess little capacity for nonplatelet lineage cell production and may therefore be “true” unipotent MkPs. Along these lines, rare young ncMkPs may represent a more quiescent cell state, possibly poised to quickly and transiently respond to stress. Indeed, we found that young ncMkPs are slower to proliferate in vitro (Figure 4C) and in vivo (Figure 5A,B), maintain higher levels of BCL‐2 (Figure 5C), and give rise to erythroid and myeloid cells upon acute need in an ablative transplantation scenario.

MkPs are predominantly GFP+ in young FlkSwitch mice (Poscablo et al. 2024; Boyer et al. 2011). Recently, stratification of young adult MkPs has been described using CD48 expression (Poscablo et al. 2024; Carrelha et al. 2024; Li et al. 2024; Morcos et al. 2022), with CD48‐ (nc)MkPs proposed to arise directly from HSCs (Carrelha et al. 2024; Li et al. 2024; Morcos et al. 2022). Although our observations partially support this view (Figure S8E–G), we cannot definitively conclude that all young ncMkPs are directly derived from HSCs given that many ncMkPs are GFP+ in FlkSwitch mice. Additionally, young ncMkPs consistently recapitulate ~20% of the total MkP pool (Figure S4B), a higher percentage than the few Tom+ MkPs (and platelets) (Poscablo et al. 2024); thus, there is likely contribution to phenotypic ncMkPs from canonical HSC differentiation (Figures 6F and 7E,F). Importantly, the impact of HSC‐direct (Tom+) MkP and platelet production in young mice is dwarfed by the enhanced platelet reconstitution capacity and resulting hyperreactive function linked to age‐dependent ncMkPs (Figure 5E–I). Thus, we have quantitatively recapitulated our original findings (Poscablo et al. 2024) in aging FlkSwitch mice and the resulting increase in age‐related platelet dysregulation and thrombosis.

Morcos et al. (2022), using an inducible labeling mouse model paired with lineage inference modeling, suggested that ~50% of all MkPs and platelets arise via an HSC‐direct pathway in young mice, a finding we and others do not recapitulate (Poscablo et al. 2024; Carrelha et al. 2024; Boyer et al. 2011). Although our MkP transplant data largely align, they reported that young CD48‐ MkPs (ncMkPs) proliferate more frequently than CD48+ MkPs (cMkPs), an observation at odds with our data (Figure 4B,C). We also demonstrate that MkP number and frequency do not necessarily correlate perfectly with peripheral blood platelet counts, as largely equivalent age‐related MkP expansion between male and female mice does not result in directly corresponding platelet increases in blood, further confounding inducible labeling studies. Similar to Morcos et al., Li et al. (2024) argued ~50% direct‐HSC contribution to the megakaryocytic pathway in young mice. They observed that ~50% of young adult MkPs are CD48‐, which we do not reproduce here (Figure S4B). Of critical importance, the inducible labeling mouse models used by Li et al. did not discriminate between HSCs and MPPs, precluding their ability to directly assess the direct HSC contribution to a parallel pathway and thus findings may be confounded by MPP involvement. A recent report by Carrelha et al. (2024) performed a series of inducible labeling and transplantations paired with scRNAseq that inferred an HSC‐to‐MkP direct path in young adult mice. However, given the additional functions of MkPs in a transplant setting (Figure 5E,F), it is possible that the experimental design confounded findings specific to the megakaryocytic lineage. They follow‐up their findings using a Flk2‐Cre lineage tracing mouse model similar to our FlkSwitch mice and concluded that ~10% of young adult platelets are produced via the HSC direct “shortcut” pathway, a low percentage closer to our observations (Poscablo et al. 2024), but not reporting or accounting for the floxing efficiency of each mouse. In the context of our data that utilizes a robust lineage tracing model paired with multiomic, phenotypic, and functional approaches, we resolve a number of these discrepancies. The direct and multifaceted observations we present here lead us to conclude that most phenotypic MkPs in young mice are generated via canonical multistep hematopoiesis and are molecularly and functionally heterogeneous, with ncMkPs specifically gaining enhanced platelet generation capacity with advancing age via direct HSC‐MkP differentiation.

Because of the prevalence of platelet‐related disorders, MkP/platelet ontogeny throughout life is an area under intense investigation (Manso et al. 2024; Poscablo et al. 2024; Carrelha et al. 2024; Li et al. 2024; Morcos et al. 2022). Encouragingly, in vitro‐derived MkPs from old HSCs recapitulate the in situ state with respect to increased numbers and altered cMkP/ncMkP frequency compared to young HSCs (Figure 6C–E). It is interesting to note that young and aged CD41‐ myeloid progenitors made few ncMkPs (Figure 6F). Aged ncMkPs (Tom+) arise directly from HSCs (Poscablo et al. 2024), while the cellular origin of young ncMkPs remains partially in question; their production may be via both canonical and noncanonical paths (Figure S8E–G). The lack of complete HSC clonal restriction to one MkP subtype indicates that environmental or other factors play a role in the MkP subtype differentiation pathway. This is consistent with the reversal of platelet lineage restriction observed previously (Carrelha et al. 2018; Yamamoto et al. 2013). Indeed, transplantation of aged HSCs into a young recipient restored young‐like ratios of cMkPs and ncMkPs (Figure 6I). It is possible that stratification methods of HSC heterogeneity, such as vWF expression (Carrelha et al. 2024), will better predict MkP subtype generation. Transplantation of single vWF+ platelet‐biased HSCs results in multilineage reconstitution (Carrelha et al. 2024), effectively “resetting” HSC function, similar to our observations (Figure 6G–I). Thus, the root cause of the age‐progressive, noncanonical MkP pathway remains elusive, possibly because of the interchangeability of stable, but reversible, states.

It was surprising to find that aging female mice had a blunted expansion of platelets compared to their male counterparts (Figure S5J–M) even though the numbers and frequencies of MkPs (and MkP subsets) are equivalent between female and male mice throughout life (Figure S5A,D,G). It is therefore tempting to speculate that sex‐based differences throughout life alter megakaryopoiesis quantitatively, but not MkP function (Figure S6A,B). While platelet hyperreactivity upon aging is conserved, in contrast to mice, humans typically have a decline in platelet count with aging (Jones 2016; Balduini and Noris 2014; Biino et al. 2011, 2013; Segal and Moliterno 2006). Although age‐associated platelet bias has been reported to be conserved between mice and humans (Aksoz et al. 2024), it remains to be seen if the canonical/noncanonical paradigm of parallel and age‐dependent mechanisms of platelet generation translate to humans and if it could be targeted to therapeutically mitigate or prevent adverse thrombotic events.

Here, we demonstrate that age‐progressive, direct HSC‐derived MkPs can be faithfully identified phenotypically and transcriptomically using the surrogate markers CD48 and CD321 to identify the analogous ncMkP (CD48‐CD321+). Aged ncMkPs recapitulate their Tom+ counterparts with striking efficiency, enabling widespread experimental application of this paradigm. We also made the surprising discovery that young mice possess rare ncMkPs that exhibit enhanced myeloid cell reconstitution capacity upon transplantation compared to their canonical counterparts, yet lack the age‐related and paradoxical increased platelet production efficiency of aged ncMkPs. Given that human HSCs gain platelet bias with age (Aksoz et al. 2024) and thrombotic diseases are major health concerns, modulating the number and type of platelets ultimately produced has huge therapeutic potential. Thus, ncMkPs may represent a foundational cellular target to explore for such modulation with the ultimate goal of alleviating patient morbidity and mortality.

## 4 Online Methods

### 4.1 Mice

All mice were housed and cared for in an AAALAC‐accredited animal facility at the University of California, Santa Cruz (UCSC) and maintained and used under approved IACUC guidelines. Barrier mouse rooms are temperature controlled and on a 12‐h day/night cycle. Mice had ad libitum access to water and food and daily care assessments. Care staff include a trained veterinarian, vivarium manager, and staff. Specific strains used for this study are C57BL/6J (wild‐type [WT], Jackson Laboratory #000664), C57BL/6‐Tg(UBC‐GFP)30Scha/J (UBC‐GFP, Jackson Laboratory #004353), and Flk2‐Cre × mT/mG mice (FlkSwitch, crossed in‐house). Unless otherwise indicated, all mice were bred and maintained in‐house. Aged WT mice were provided by the National Institutes of Health Rodent Ordering System. All mouse ages (in months) and numbers are indicated in figure legends; young are 2 to 3 months old and aged are 20 to 24 months old. WT and UBC‐GFP mice were randomized based on sex, whereas male FlkSwitch mice were used.

### 4.2 Mouse Tissue Collection and Processing

When bone marrow was collected, mice were euthanized via CO_2_ inhalation as mandated by the approved UCSC IACUC protocols, followed by cervical dislocation. Hind legs, hips, forelegs, and/or sternum were dissected, cleaned, and immediately placed in staining media (1X PBS [cytiva], 5 mM EDTA [Millipore Sigma], and 2% bovine calf serum [BCS, cytiva]) on ice. If cell populations were quantified, a known number of APC Calibrite beads (BD Biosciences) were added immediately prior to processing. Bones were processed either by crushing via mortar and pestle or spin‐isolated in staining media (Heib et al. 2021). Following cell release from bones, cells were passed through a 70 μm filter and centrifuged for 5 min at 300× *g*. Peripheral blood was collected via nicking the tail vein unless otherwise indicated. A known volume of blood was immediately added to staining media containing a known number of APC Calibrite beads (BD Biosciences) and appropriate antibodies for flow cytometry analysis (see Flow Cytometry methods).

### 4.3 Hematopoietic Stem and Progenitor Cell Enrichment

Hematopoietic stem and progenitor cells were enriched from freshly isolated whole BM via positive magnetic selection. Cells were suspended in 0.5 to 1.0 mL staining media and incubated with 2 μL/bone anti‐CD117 (cKit) magnetic beads (Miltenyi Biotec) for 30 min on ice, mixing at the 15 min mark. Positive selection via LS columns (Miltenyi Biotec) was performed according to the manufacturer's instructions. The cKit‐enriched cell fraction was then washed in staining media and used immediately.

### 4.4 Flow Cytometry and Cell Sorting

#### 4.4.1 Whole or cKit‐Enriched BM

Cells to be stained were aliquoted into either 5 mL polystyrene tubes or individual wells of a 96‐well U‐bottom plate. Cells were then blocked for a minimum of 10 min on ice in a solution of staining media with 5% each normal mouse and rat serum (Invitrogen). If the experimental design used unconjugated mouse‐derived antibodies (and antimouse secondary), only those were used first, followed by blocking. The appropriate antibody cocktail was then added directly to cells plus block, and incubated for 30 min on ice in the dark. Cells were then washed and either stained with streptavidin secondary for 20 min on ice in the dark or resuspended for use. Cells were thoroughly washed with staining media between each staining step. Immediately prior to acquisition, 3 drops of 1:5000 diluted propidium iodide (PI, MP Biologicals) were added and cells were mixed. Where indicated, negative controls (fluorescent minus one or isotype) were employed.

#### 4.4.2 Peripheral Blood

The primary antibody cocktail was prepared in staining media together with a known number of APC Calibrite beads (BD Biosciences) and placed on ice. A known volume of blood was collected (typically 10 μL) and immediately mixed well in the antibody/bead solution. Cells were then treated as described for BM above. Following completion of staining, ~10% of the homogenized and stained blood was aliquoted and diluted for whole blood (platelet and erythrocyte) analysis. The remaining sample was subjected to erythrocyte lysis via ammonium‐chloride‐potassium (ACK, 0.15 M NH_4_Cl, 1 mM KHCO_3_, and 0.1 mM Na_2_EDTA in diH_2_O, pH 7.2) incubation. Cells were washed, pelleted, and resuspended in 1X ACK at room temperature for 5 min. Cells were then washed with staining media. Immediately prior to acquisition, 3 drops of PI solution were added followed by cell mixing.

#### 4.4.3 In Vitro Analysis

At termination of in vitro cultures, a known number of APC Calibrite beads (BD Biosciences) was added to each well. Plates were then centrifuged for 5 min at 300xg (~1250 RPM). Supernatant was manually removed via pipetting, and samples were blocked and stained as described above. To preserve rare cell numbers, cells were not washed but resuspended in 1:5000 diluted PI following completion of staining.

#### 4.4.4 CellTrace Violet (CTV) Staining

Freshly isolated BM from young and aged mice was prepared and cKit‐enriched as described above. Following antibody staining, the enriched fraction was then labeled with CTV following the manufacturer's instructions. The highest CTV‐labeled cells were then isolated via fluorescence‐activated cell sorting (FACS), selecting for strong and uniform CTV signal by double‐sorting each population, and subsequently cultured in vitro.

#### 4.4.5 Flow Cytometric Viability Assessment

Freshly isolated BM or in vitro culture endpoint samples were stained with antibodies for analysis as described above, except for the use of the Annexin V binding buffer (BioLegend) instead of staining media. Ghost Dye Violet 510 fixable viability dye (FVD, Cytek) was included simultaneously with antibody staining at a final concentration of 1:1000. Following staining, Annexin V and 7‐AAD (BioLegend) were added for 15 min at room temperature in the dark as per the manufacturer's instructions. Annexin V binding buffer was then added, and samples were immediately acquired.

#### 4.4.6 Ki‐67 Staining

Cell‐surface antibodies were used as described above. Cells were then fixed, permeabilized, and stained with the FoxP3 kit (BioLegend) per manufacturer's instructions. An isotype control of equal protein amount was used to determine percent Ki‐67 positive cells. Following completion of staining, cells were resuspended in 1% PFA (Electron Microscopy Sciences) in 1X PBS for analysis.

#### 4.4.7 MkP Ploidy Assessment

Following preparation of BM single‐cell suspensions, lineage‐positive cells were depleted by first labeling with rat‐antimouse lineage‐specific antibodies (CD4, CD8, CD3, CD11b, B220, CD5, and Gr‐1, all BioLegend) for 20 min on ice. Following washing, prewashed sheep‐anti‐rat Dynabeads (Invitrogen) were added to cells on ice for 20 min, gently mixing every 5 min. The labeled cells+beads were then placed in a magnet (StemCell Technologies) for 2 min, followed by supernatant decantation to collect the lineage‐negative fraction. Fresh beads were then added to the initial lineage‐negative fraction for 10 min, followed by an additional negative selection step as described above. Cells were then stained with goat‐anti‐rat secondary and additional cell surface staining steps as described above. Following washing, cells were incubated with Hoechst 33342, trihydrochloride trihydrate (ThermoFisher Scientific) at a concentration of 2 μg/mL for 1 h at 37°C (water bath). Cells were washed twice and immediately ran on the flow cytometer.

#### 4.4.8 In Vivo 5‐Ethynyl 2′‐Deoxyuridine (EdU) Incorporation and Detection

EdU was dissolved in 1X PBS and injected intraperitoneal (i.p.) at a concentration of 50 mg/kg mouse weight. Twenty‐four hours post‐injection, mice were sacrificed and BM prepared as described above. Detection of EdU was performed via the Click‐iT Plus EdU Flow Cytometry Assay Kit (Invitrogen) per manufacturer's instructions. Negative control mice were EdU‐injected, but did not undergo the Alexa Fluor 647 labeling step.

#### 4.4.9 BCL‐2 Staining

Cell‐surface antibodies were used as described above. Cells were then fixed, permeabilized, and stained with the True‐Nuclear kit (BioLegend) per manufacturer's instructions. An isotype control of equal protein amount was used to determine relative BCL‐2 expression following standardized flow cytometry approaches (Manso and Medina 2021). Following completion of staining, cells were resuspended in 1% PFA (Electron Microscopy Sciences) in 1X PBS for analysis.

#### 4.4.10 Platelet Activation Assay Using Washed Platelets in Whole Blood

Whole blood (20 μL) was collected from young or aged FlkSwitch mice via retro‐orbital bleeding using EDTA‐coated capillaries (Sarstedt). Blood was immediately transferred into 300 μL porcine heparin (0.3 mg/mL, Fisher Scientific) prepared in 1X PBS. Samples were washed twice with Tyrode's buffer (2 mL; 800 ×*g*, 5 min, room temperature). The Tyrode's buffer consisted of: 137 mM NaCl, 0.3 mM Na_2_HPO_4_, 2 mM KCl, 12 mM NaHCO_3_, 5 mM HEPES, 5 mM glucose, and 0.35% BSA (made in‐house) and was prepared without CaCl_2_ for the initial washes. Washed platelets were then resuspended in Tyrode's buffer with calcium (supplemented with 2 mM CaCl_2_). Platelets from young FlkSwitch mice were stained with antimouse CD41‐Brilliant Violet 421 (BioLegend) for 30 min at room temperature in the dark. Platelets from aged mice were stained with antimouse CD41‐Alexa Fluor 700 (BioLegend). Both young and old samples were costained with antimouse Ter119 PE‐Cy5 (BioLegend) to exclude erythroid‐lineage cells. After staining, 5 × 10^5^ CD41+Ter119‐ platelets from young and 5 × 10^5^ from aged mice were combined into Tyrode's buffer containing 2 mM CaCl_2_ and anti‐P‐selectin (CD62P)‐PE‐Cy7 (BioLegend). Baseline (resting) platelet state was acquired for 5 min in the absence of any activator. Platelet activation was then induced with either human thrombin (0.1 U/mL, Sigma‐Aldrich) or ADP (10 μM, Sigma‐Aldrich) in separate samples. Immediately following activation, samples were acquired by flow cytometry for 5 min at a flow rate of 10 μL/min using a CytoFLEX LX (Beckman Coulter). Platelet activation was assessed by quantifying P‐selectin neo‐exposure over time. Data were analyzed using FlowJo (BD Biosciences), calculating area under the curve (AUC) for P‐selectin neo‐exposure in GFP+ or Tom+ CD41+Ter119‐ washed platelets in whole blood.

#### 4.4.11 Cytometers

FACS was performed via a BD Biosciences FACSAria IIu. Cell analysis was similarly performed on the FACSAria or Cytoflex LX (Beckman Coulter). All flow cytometry and FACS data was analyzed via FlowJo (BD Biosciences). The FlowJo plugin IndexSort was used for analysis of single sorted HSCs.

#### 4.4.12 Phenotypic Cellular Definitions and Example Flow Cytometry Staining

Figure S11 details all cellular definitions used and illustrates representative flow cytometry data for each cell type and experimental approach.

### 4.5 Single Cell RNAseq Analyses

We performed pseudobulk differential expression analysis using the Python3 implementation of DESeq2 (Love et al. 2014; Muzellec et al. 2023) as previously performed (Poscablo et al. 2024). Briefly, we restructured the single‐cell dataset into sample‐level datasets. We extracted the sample and cell type clusters for young GFP+, old GFP+, and old Tom+ MkPs and aggregated our scRNAseq data into pseudobulk samples by summing the counts of individual cells within each biosample and condition. We ran DESeq2 on the pseudobulked count matrix to generate a list of differentially expressed genes between two conditions of interest. We also computed the baseMean of each gene within a condition by taking the mean normalized counts of a gene across cells within that condition. The DEG lists were then further filtered by removing mitochondrial and ribosomal gene transcripts, as well as Malat1 transcripts, to reduce technical bias, as well as those whose baseMean values were less than 5 in both conditions or had no calculated adjusted *p* value. We subsequently conducted a GO enrichment analysis using DEGs generated from pseudobulk DEseq2 output using GOATOOLS. GO Terms reported required at least 20% DEG representation for each GO Term. The scores of cell cycle phases were calculated using the Scanpy function “score_genes_cell_cycle” on the basis of canonical markers (Kowalczyk et al. 2015).

### 4.6 Cellular Indexing of Transcriptomes and Epitopes Sequencing (CITEseq)

#### 4.6.1 Sample Preparation

CITEseq via the 10X Genomics platform was performed simultaneously with our previously published scRNAseq data of young and aged FlkSwitch mice (Poscablo et al. 2024). Briefly, cKit‐enriched BM was sorted into CD150+ and CD150‐ fractions and incubated with CITEseq antibodies (BioLegend) per manufacturer's instructions. CD321 was not included as it was not available. All other steps as described in Poscablo et al. 2024. Chromium Next GEM Single Cell 3′ v3.1 (Dual Index) reagents and protocols were utilized as specified. Sequencing depth of 5000 read pairs per cell was performed.

#### 4.6.2 CITEseq Data Preparation and QC

Cells expressing fewer than 200 genes were excluded from the analysis. Cells were further filtered based on surface protein marker expression; specifically, double‐negative cells for CD117 antibody‐derived tag (ADT) and *Kit* RNA were excluded, removing 13 cells from the dataset. To normalize the CITEseq data, we implemented a centered log‐ratio (CLR) transformation for each cell (Stoeckius et al. 2017). For each cell's ADT count vector, we first computed the natural logarithm of the counts, offset by one, for all nonzero elements. The sum of these log‐transformed values was then divided by the total number of features, and the exponential of this mean was used to normalize the original counts. Finally, a log transformation was applied to the resulting normalized values to stabilize variance across features.

#### 4.6.3 Differential Protein Expression Analysis

We quantitatively compared ADTs generated by CITEseq using the sc.rank_genes_group function that uses the *t*‐test_overestim_var method in the Scanpy python package. Although included in the analysis, lineage (CD3, CD4, CD8a, B220, Gr1, Ter119, and CD11b) and MkP‐defining (CD117/cKit, Sca1, CD150, CD41) markers were excluded from the display of Figure 1E.

#### 4.6.4 Additional Annotations

We generated three annotations of interest:

1. RNA + Protein: RNA annotated MkPs (Poscablo et al. 2024) further split into cMkP (CD48+) and ncMkP (CD48‐) via CITEseq CD48 expression (Figure S3A).
2. Protein only: CITEseq‐only annotation of cMkPs (CD48+) and ncMkPs (CD48‐), mimicking flow cytometric‐based cell subsetting (Figure S3B).
3. RNA only: RNA‐based classification using *Cd48* and *F11r* (CD321) expression utilizing defined MkPs (Poscablo et al. 2024). Cells were evaluated for *Cd48* and *F11r* expression and classified as either *Cd48* +*F11r*‐ or *Cd48*‐*F11r* + (Figure S3C). We excluded 98 cells that had both *Cd48* and *F11r* expression, as well as 481 cells that had no *Cd48* or *F11r* expression.

Venn Diagrams of annotation version comparisons generated via the R package Vennerable, with input as described in the text and figure legends.

Each annotation set was further divided by sample age (young and old). RNA‐based annotations were previously done by Poscablo et al. 2024 where hematopoietic cell states were annotated using only scRNAseq data. For the CD48 protein call of positivity (annotations (1) and (2) above), we applied a two‐component Gaussian Mixture Model (GMM) via the GaussianMixture function in Scikit‐learn (Pedregosa et al. 2011) to CD48 protein expression among cells annotated as MkP via RNA expression. From the inferred mean values of the two components, we derived the CD48 positive and negative group threshold as the mean of these components: 1.1. For annotation (2), we manually derived thresholds for CITEseq proteins of interest to annotate MkPs via protein expression. MyPros were annotated at expression values of Sca‐1 < 0.71 and CD117 > 1. We subsequently annotated these myeloid progenitor cells as MkPs if they had CD150 > 0.45 and CD41 > 0.6 expression. Lastly, these MkP CITE‐annotated cells were classified as CD48+/− by applying the previously determined threshold of 1.1.

#### 4.6.5 Selection of Cell Surface MkP Candidates via Bulk RNAseq and scRNA/CITEseq

Bulk RNAseq comparisons of aged GFP+ and Tom+ MkPs as described previously (Poscablo et al. 2024) were deeply analyzed. First, DEG lists were filtered to remove any genes with an average read count of 50 or less across both cell states, as well as those with no computed adjusted *p* value. Next, we applied two possible thresholds to identify genes with the largest differences: a strict threshold consisting of genes with a Log2FoldChange ≥ 2.5 and adjusted *p* value < 0.01, and a relaxed threshold of Log2FoldChange ≥ 1.5 or adjusted *p* value < 0.1. Finally, genes were further filtered to select for those predicted to be membrane‐bound, extracellularly expressed, and with commercially available antibodies against an individual gene's protein product. The scRNAseq data was similarly treated (see above) following pseudobulking (Poscablo et al. 2024), with the only exception of removing any genes with an average read count of 5 or less across both cell states. We utilized pseudobulked data to best identify targets that enrich for the MkP subpopulations without biasing for expression outliers (Squair et al. 2021). Volcano plots of DEGs were generated via the EnhancedVolcano package in R, with labels assigned as detailed in figure legends.

CITEseq candidates were assessed via Wilcoxon rank score comparing aged GFP+ and Tom+ MkPs. Protein targets of mature lineage cells (CD3, CD4, CD5, CD8a, CD11b, Gr1, B220, and Ter119) were not included in the analysis. Similarly, protein targets used to define the MkP cell state (cKit/CD117, Sca‐1, CD150, and CD41) were not included. A restrictive Wilcoxon score cutoff of 10 and −10 was applied, as well as a relaxed threshold of 5 and −5.

#### 4.6.6 Bulk In Vitro Cultures

HSCs, CD41‐ MyPros, cMkPs, and ncMkPs were isolated via FACS from fresh BM as described above. All populations were double‐sorted to ensure purity. Sorted cells were cultured in a 96‐well U‐bottom plate and incubated at 37°C, 5% CO_2_. 200 HSCs, 2000 CD41‐ MyPros, 2000 cMkPs, or 2000 ncMkPs were plated per well, and up to three replicates were set up per mouse and cell type. HSCs and CD41‐ MyPros were cultured for 7 days in IMDM plus GlutaMAX (Gibco) containing 20% fetal bovine serum (FBS, Atlas Biologicals), 1X Primocin (InvivoGen), 1X nonessential amino acids (Gibco), and recombinant mouse thrombopoietin (TPO, 50 ng/mL), stem cell factor (SCF, 20 ng/mL), IL‐6 (20 ng/mL), IL‐3 (10 ng/mL), and IL‐11 (20 ng/mL, all Peprotech). MkP subpopulations were cultured for 3 days in IMDM plus GlutaMAX containing 10% FBS, 1X Primocin, 1X nonessential amino acids, and recombinant mouse TPO (20 ng/mL), SCF (50 ng/mL), IL‐6 (20 ng/mL), and IL‐3 (5 ng/mL) as previously described (Poscablo et al. 2024, 2021).

### 4.7 Indexed Single Cell HSC In Vitro Cultures

Single HSCs were prepared, isolated, cultured, and analyzed as described above, with the exception of using the BD FACSDiva single‐cell sorting software module per manufacturer's directions, with the index sorting option selected.

### 4.8 MkP Transplantations

cMkPs and ncMkPs from young and aged mice were freshly isolated via FACS from cKit‐enriched BM as described above. Populations were sorted twice to ensure specificity. For each individual transplant, 22,000 MkPs were injected retro‐orbitally in 50 μL 1X HBSS (Corning). 1X HBSS only sham controls were also utilized. Recipient UBC‐GFP mice were preconditioned by same‐day sublethal irradiation (500 rads) and anesthetized via isoflurane (MWI Veterinary Supply Company) for injections. Peripheral blood was assessed as described above. Donor‐derived chimerism was determined as the frequency of GFP‐ cells among a given cell population, with the baseline frequency background‐subtracted. Similarly, cell number was determined via quantification of GFP‐(donor‐derived) cells as described above, background subtracted against the baseline number.

### 4.9 HSC Transplantations

HSCs from young and aged mice were freshly isolated via FACS from cKit‐enriched BM as described above. Populations were sorted twice to ensure specificity. For each individual transplant, 200 HSCs were injected retro‐orbitally in 50 to 100 μL 1X HBSS (Corning). Recipient mice were preconditioned by same‐day sublethal irradiation (500 rads) and anesthetized via isoflurane (MWI Veterinary Supply Company) for injections. After 16 to 20 weeks of reconstitution, BM was harvested and assessed via flow cytometry as described above. Donor‐derived chimerism was determined as the frequency of fluorescent cells among a given cell population. Cell numbers were similarly determined as described above.

List of reagents and software can be found in Table S5.

### 4.10 Statistical Analysis

Statistical tests are indicated in figure legends and performed using GraphPad Prism. For all tests, *p* values are indicated numerically or otherwise displayed as *p < 0.05, **p < 0.01, ***p < 0.001, or ****p < 0.0001, and “ns” (or lack of designation) indicates nonstatistical significance. Paired *t*‐tests, unpaired *t*‐tests, and Spearman correlations are two‐tailed. Multiple unpaired *t*‐tests were performed with Holm‐Šídák multiple comparisons adjustment. Unless otherwise indicated, graphs of summary statistics indicate the mean ± standard deviation (SD). The number of individual experiments and samples is outlined in the figure legends.

## Author Contributions

B.A.M. and E.C.F. conceived of the study. B.A.M. wrote the manuscript with input from all authors. P.M. performed the bioinformatics analyses with input from L.M. and B.A.M. with oversight from V.D.J. B.A.M., S.S.‐B., A.R.B., E.B., A.D., S.B.A., C.V.V., S.C., M.G.E.R., and J.M. performed all experiments and data analysis. All authors reviewed and contributed to manuscript revisions.

## Conflicts of Interest

The authors declare no conflicts of interest.

## Supporting information


**Data S1:** acel70221‐sup‐0001‐DataS1.zip.

## Data Availability

All sequencing data is available via the NCBI GEO Accession repository. Bulk RNAseq data was previously published (Poscablo et al. 2024) (GSE226318). scRNAseq data was previously published (Poscablo et al. 2024) (GSE255019). scCITEseq data is available via GSE280357. Note that the combined scRNA/CITEseq data can be accessed via the GSE280510 accession super series. The data that support the findings of this study are openly available in GEO at https://www.ncbi.nlm.nih.gov/geo/. No custom code was generated for this research.
