# Supplementary material for: A Rare Hematopoietic Stem Cell‐Derived Megakaryocyte Progenitor Accumulates via Enhanced Survival and Contributes to Exacerbated Thrombopoiesis Upon Aging
Source: Aging Cell. 2025 Sep 23;24(11):e70221. doi: 10.1111/acel.70221 (PMC12611322; doi:10.1111/acel.70221)
Supplement: Supplementary file 1 — Data S1: acel70221‐sup‐0001‐DataS1.zip. [file ACEL-24-e70221-s001.zip › acel70221-sup-0001-DataS1/Manso Aging Cell Supplemental Info R1.3.docx]

**Supplemental Information**

**A rare hematopoietic stem cell-derived megakaryocyte progenitor accumulates via enhanced survival and contributes to exacerbated thrombopoiesis upon aging**

Bryce A. Manso, Paloma Medina, Stephanie Smith-Berdan, Alessandra Rodriguez y Baena, Elmira Bachinsky, Lydia Mok, Angela Deguzman, Sarah Beth Avila, Connor Van Voorhis, Saran Chattopadhyaya, Marcel G.E. Rommel, Jenna Myers, Vanessa D. Jönsson, and E. Camilla Forsberg.

Supplemental Figures 1-11

Supplemental Figure legends 1-11

Submitted in conjunction with Supplemental Tables 1-5

**Supplemental Figure 1**

**
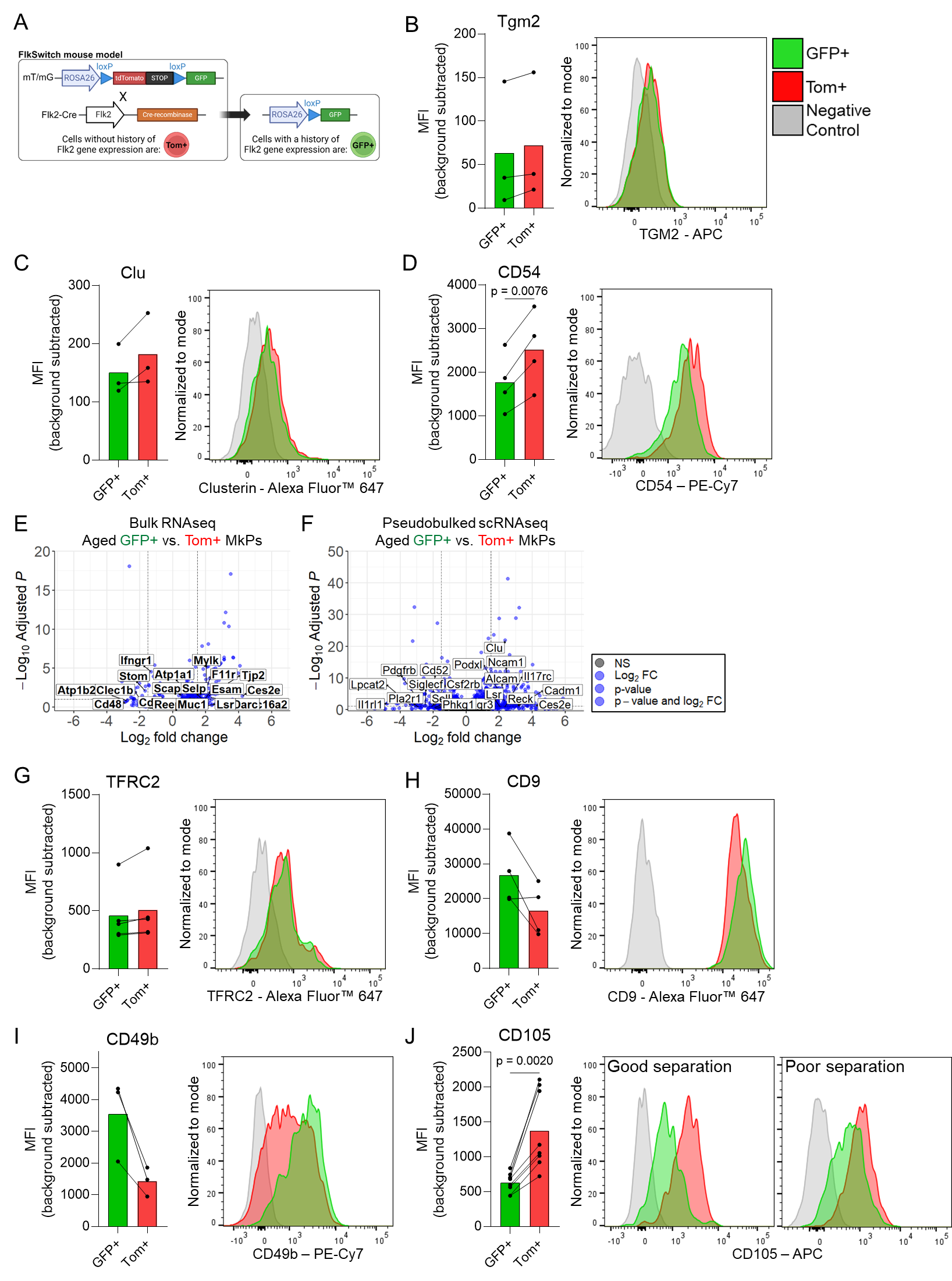
**

**Supplemental Figure 1. Transcriptomic and phenotypic heterogeneity of aged GFP+ and Tom+ MkPs.**

**A.** Schematic of FlkSwitch mouse model transgenes.

**B-D and G-J.** Flow cytometry analysis for the expression of the indicated marker on aged GFP+ and Tom+ MkPs from FlkSwitch mice. Each point represents an individual mouse with lines connecting values from the same mouse. Example histograms demonstrating staining pattern. n=3-8 across 8 independent experiments.

**E-F.** Volcano plots of **E.** bulk and **F.** scRNAseq data comparing aged FlkSwitch GFP+ and Tom+ MkPs from Poscablo et al., 2024^1^. Thresholds are adjusted p-value <0.1 and absolute log_2_ fold change ≥1.5, indicated by blue points. Labeled genes indicate those predicted to encode cell surface proteins that have commercially available flow cytometry compatible antibodies. Only the top 10 genes in each direction by log_2_ fold change displayed for graphical simplicity, excluding those indicated in Figure 1C-D. See Table S1 for complete gene lists. **E.** 8120 total genes and **F.** 11570 total genes plotted.

**Supplemental Figure 2**


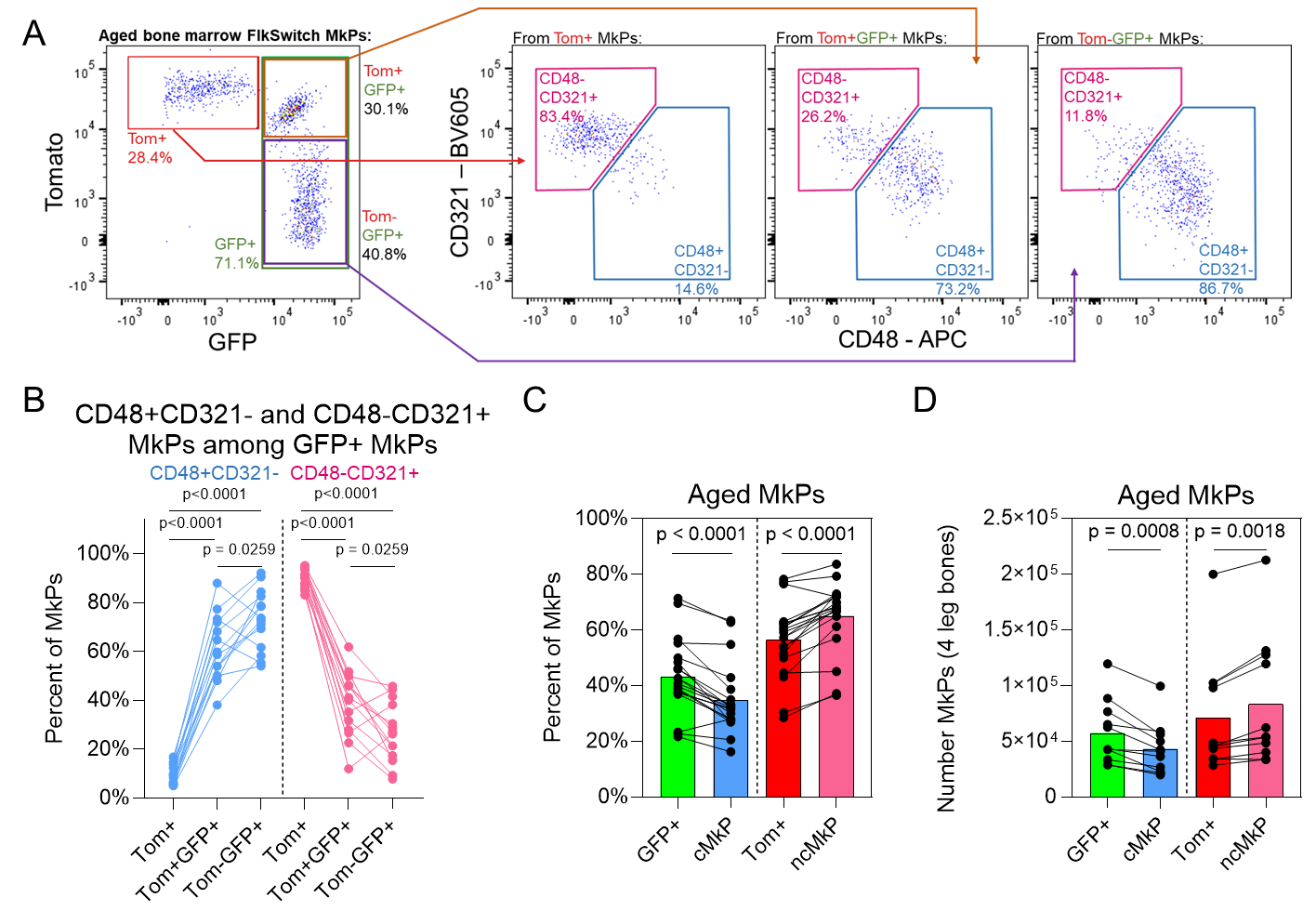


**Supplemental Figure 2. Inter-mouse assessment of aged MkP subpopulations.**

**A.** Example flow cytometry plots of CD48+CD321- and CD48-CD321+ MkPs among Tom+(GFP-), Tom+GFP+, and Tom-GFP+ aged FlkSwitch MkPs. **B.** Comparison of the frequency of CD48+CD321- and CD48-CD321+ MkPs among Tom+GFP-, Tom+GFP+, and Tom-GFP+ aged FlkSwitch MkPs. Each point represents an individual mouse with lines connecting populations from the same mouse. n=14 mice across 8 independent experiments. Note that not all aged mice possess a Tom+GFP+ MkP population, which were excluded from this specific analysis. Statistical significance determined by repeated measures one-way ANOVA adjusted for multiple comparisons (Tukey). **C-D.** The **C.** frequency and **D.** number of aged MkP populations, comparing GFP+ to cMkP and Tom+ to ncMkP via paired analyses (same data as in Figure 2C-D). Each point indicates an individual animal with lines connecting the same mice. **C.** n=20 across 12 independent experiments and **D.** n=11 across 5 independent experiments.

**Supplemental Figure 3**

**
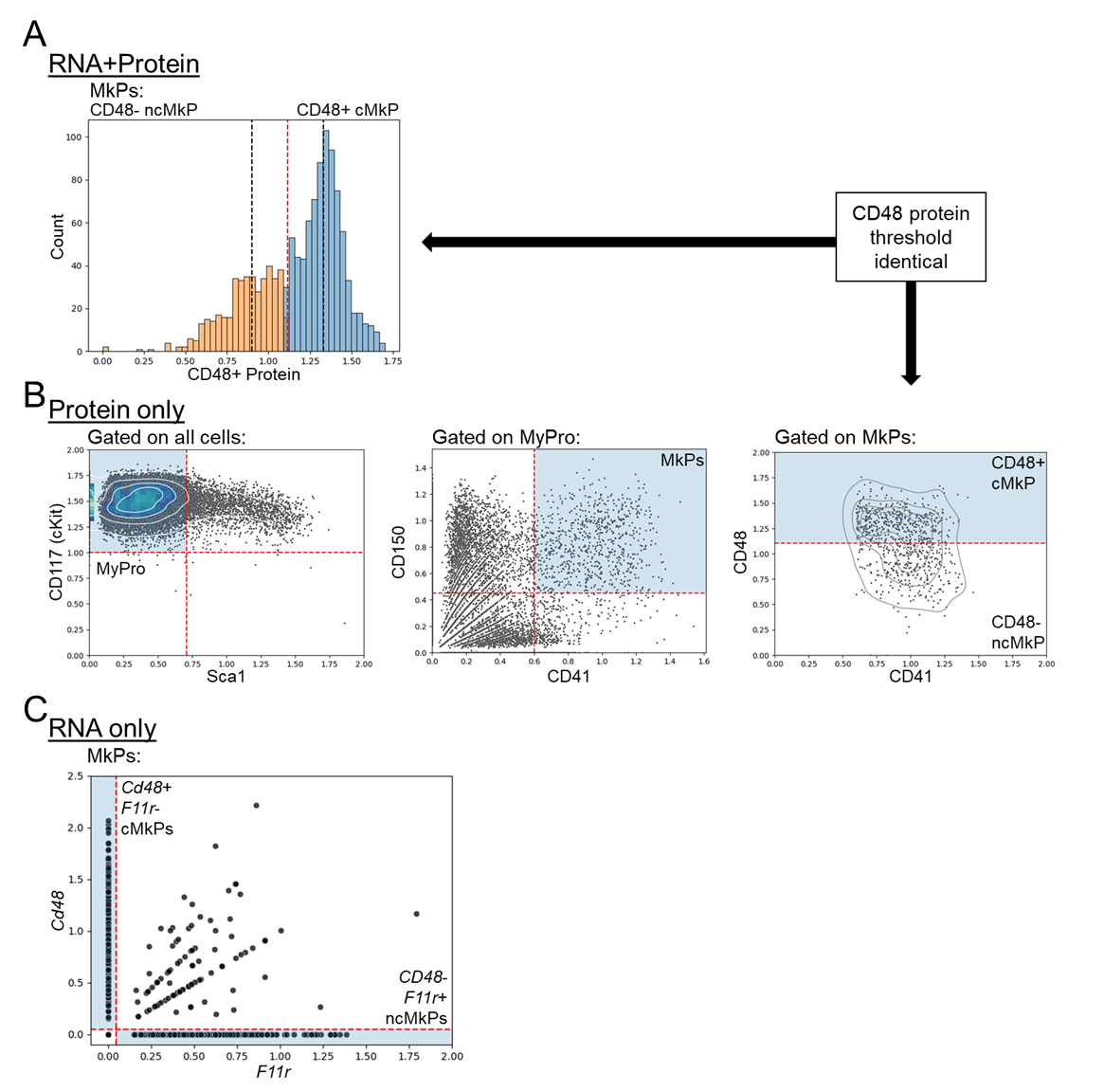
**

**Supplemental Figure 3. Additional scRNA/CITEseq analysis of aged FlkSwitch bone marrow MkPs.**

**A-C.** Additional details in the Methods.

**A.** Histogram showing CD48 protein expression among all scRNAseq-annotated MkPs. Gaussian mixture model was used to classify MkPs as cMkPs and ncMkPs based on CD48 protein expression. Black dashed line is the median of the cMkP and ncMkP CD48 distributions, while the red dashed line is the mean of the two medians.

**B.** Protein gating strategy of scCITEseq data to annotate MyPro and MkP populations similar to flow cytometry. Red dashed lines and blue shaded boxes show thresholds used to annotate cells.

**C.** Annotation of cMkP and ncMkP via *F11r* and *Cd48* RNA expression among all scRNAseq annotated MkPs. *Cd48*+*F11r*- represent cMkPs whereas *Cd48*-*F11r*+ indicate ncMkPs. Red dashed lines and blue shaded boxes indicate thresholds used to annotate cells.

**Supplemental Figure 4**

**
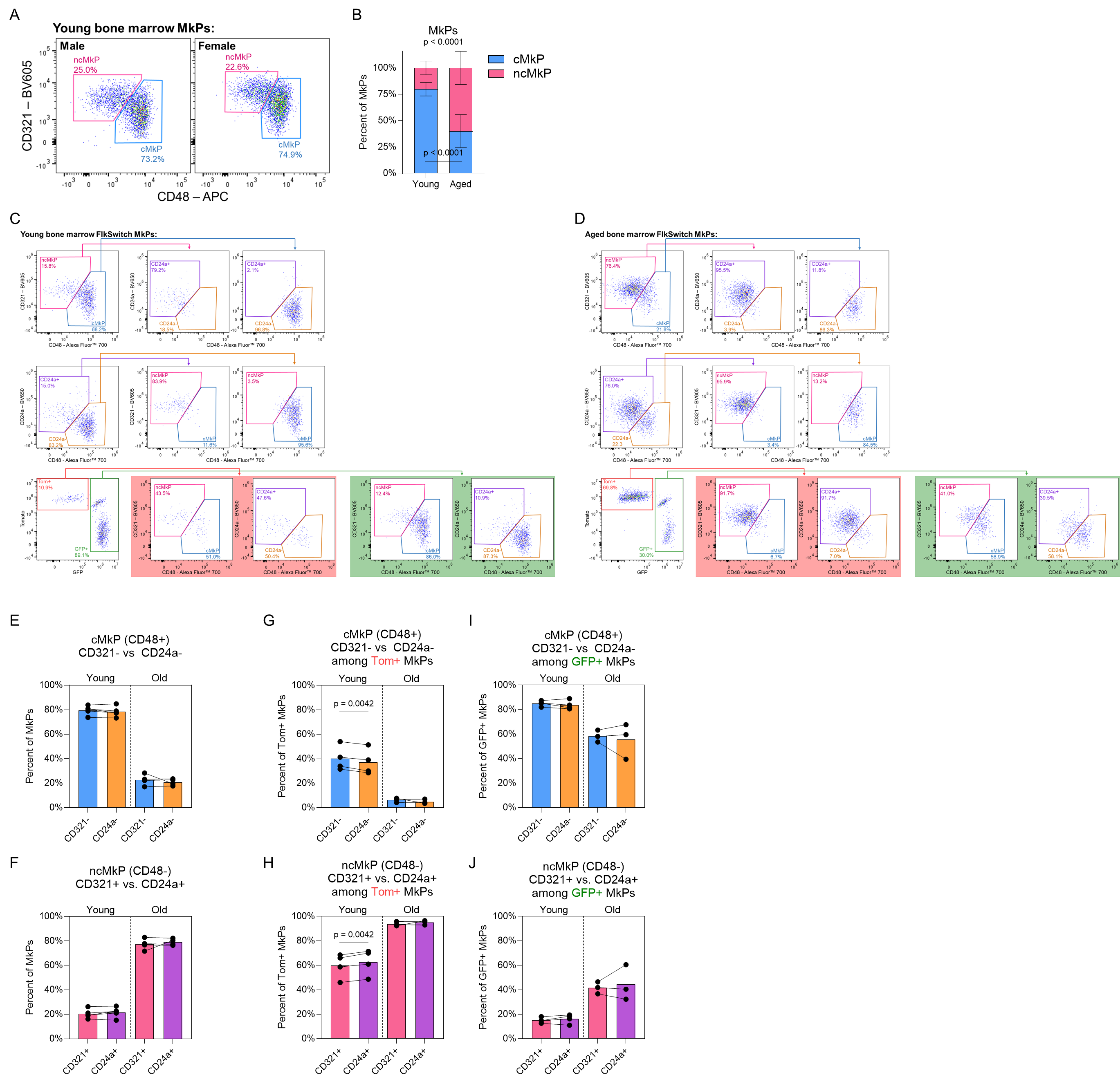
**

**Supplemental Figure 4. Investigation of young MkP phenotypic heterogeneity.**

**A.** Example flow cytometry plots demonstrating detectable, yet rare, ncMkPs in young mice.

**B.** Comparison of the cMkP and ncMkP frequency between young and old WT mice. n=39 and old n=43 mice across more than 10 independent experiments.

**C-J.** Evaluation of the potential for the cell surface marker CD24a, similar to CD321, to enrich for ncMkPs among Young Aged FlkSwitch mice. **C-D.** Example flow cytometry plots. **E-F.** Comparison of MkP subset frequency using either CD321 or CD24a in conjunction with CD48. **G-J.** Comparison of MkP subset frequency using either CD321 or CD24a in conjunction with CD48 among either **G-H.** Tom+ or **I-J.** GFP+ MkPs. **E-F.** n=4 across 3 independent experiments. **G-J.** n=3 across 2 independent experiments. **E-J.** Individual mice are represented by a single point, with lines connecting data from the same mouse. Statistical analyses are 2-tailed paired t-tests.

**Supplemental Figure 5**

**
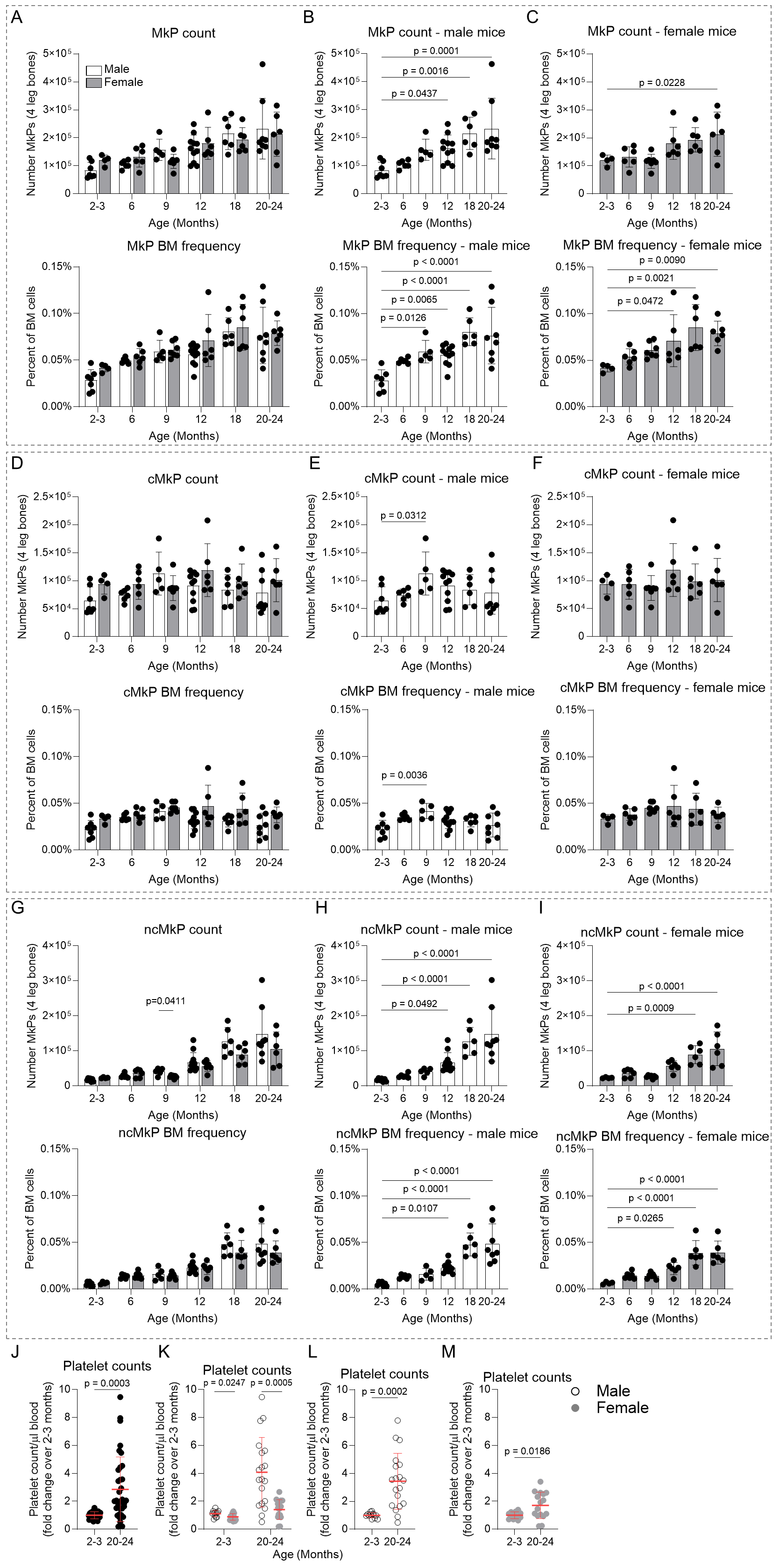
**

**Supplemental Figure 5. Assessment of megakaryopoiesis by sex throughout life.**

**A-I.** Quantification (top) and frequency (bottom) of total BM flow cytometry analysis of BM MkPs from male and female mice. Each point represents an individual mouse. n=11, 12, 12, 17, 12, and 14 for ages 2-3, 6, 9, 12, 18, and 20-24 months, respectively across 10 independent experiments.

**A, D, G.** Comparison of **A.** total MkPs, **D.** cMkPs, and G**.** ncMkPs between male and female mice across six age groups. *p<0.05 by multiple unpaired t-tests with Holm-Šídák multiple comparisons adjustment.

**B-C, E-F, G-I.** Comparison of male and female **B-C.** total MkPs, **E-F.** cMkPs, and **G-I.** ncMkPs numbers over time. One-way ANOVA, comparing all groups to the 2-3 month age group by Dunnett’s multiple comparisons test.

**J-M.** Total platelet counts/μl blood as determined by quantitative flow cytometry, displayed as fold change over 2-3 month old mice. Each point represents an individual mouse and error bars are mean ± SD. n=24 young and n=35 old across 5 independent experiments.

**Supplemental Figure 6**


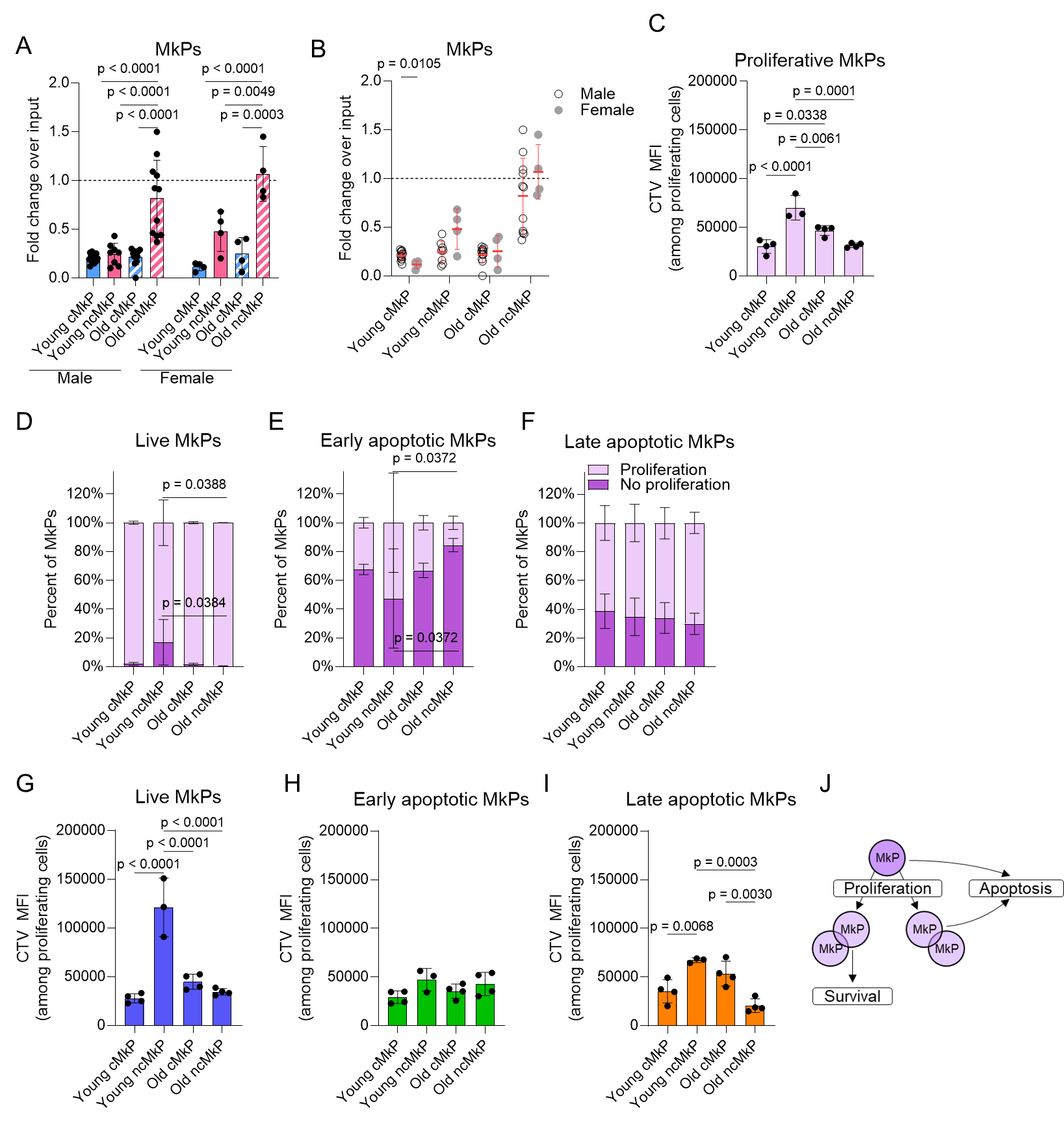


**Supplemental Figure 6. ncMkPs have a survival advantage in vitro.**

**A-B.** Comparison within **A.** or between **B.** MkPs from male and female mice. n and experimental design as in Figure 4A. **A.** One-way ANOVA adjusted for multiple comparisons via Tukey’s test or **B.** multiple unpaired t-tests (similarly adjusted for multiple comparisons).

**C.** MFI of CTV among proliferating MkPs.

**D-F.** Proportion of in vitro cultured MkPs that proliferated over the three-day culture, stratified by viability cell state.

**G-I.** MFI of CTV among proliferating MkPs of the indicated viability states.

**C-I.** n=3-4 across 4 independent experiments. Up to three technical replicates per mouse were averaged together as cell number allowed. One-way ANOVA adjusted for multiple comparisons via Tukey’s test. **D-F.** Data stacked for visual simplicity.

**J.** Model of in vitro MkP proliferative and survival performance.

**Supplemental Figure 7**


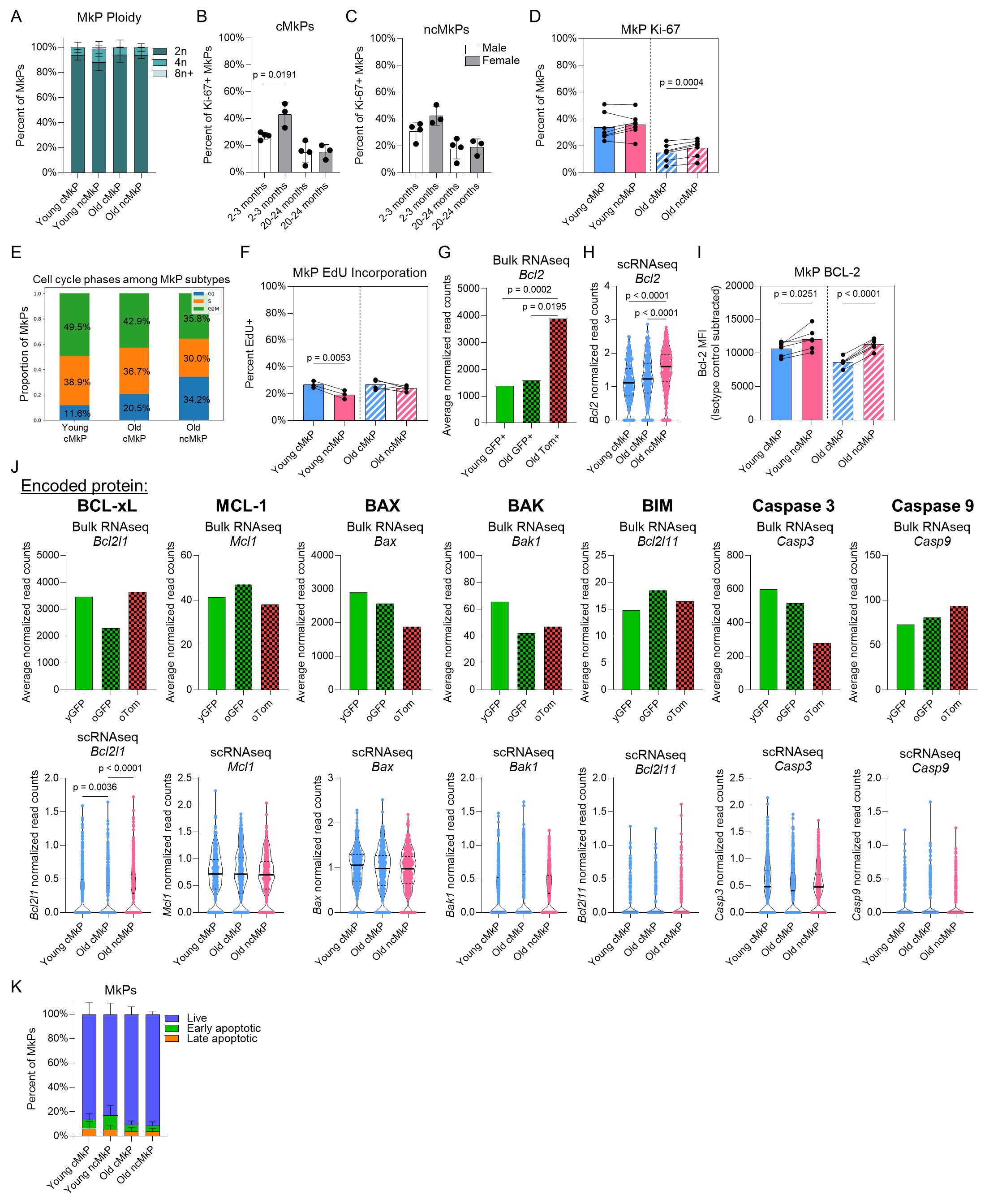


**Supplemental Figure 7. In situ features of MkP subtypes.**

**A.** Ploidy analysis from freshly isolated BM reveals <1% 8n+ cells among phenotypic MkPs in both young and old mice.

**B-D.** Frequency of Ki-67+ cells among young and old MkP subtypes from freshly isolated BM. **B-C.** Comparison between male and female mice. **D.** Comparison within mice. Each point represents a single mouse with lines connecting cells from the same mouse. n and experimental design as in Figure 5A.

**E.** Proportion of MkP cell cycle phases inferred from scRNAseq signatures. Young ncMkPs omitted due to low cell numbers.

**F.** Comparison of 24 hour EdU incorporation within individual mice. Each point represents a single mouse with lines connecting cells from the same mouse. n and experimental design as in Figure 5B.

**G-H.** Read count data of *Bcl2* from **G.** bulk and **H.** scRNAseq. Statistical significance determined via DESeq2 comparisons^1,2^ for **G.** and by one-way ANOVA adjusted for multiple comparisons via Tukey’s test for **H.**

**I.** Paired analysis within individual mice for relative abundance of BCL-2 protein. n and experimental design as in Figure 5C.

**J.** Read count data of several apoptosis and stress pathway genes from bulk (top row) and scRNAseq (bottom row). Analysis and statistical testing as in **G-H.**

**K.** Proportion of viability cell states among freshly isolated MkPs. n=6-8 across 4 independent experiments. Statistical non-significance determined by one-way ANOVA adjusted for multiple comparisons via Tukey’s test, each cell state tested separately. Data stacked for visual simplicity.

**Supplemental Figure 8**


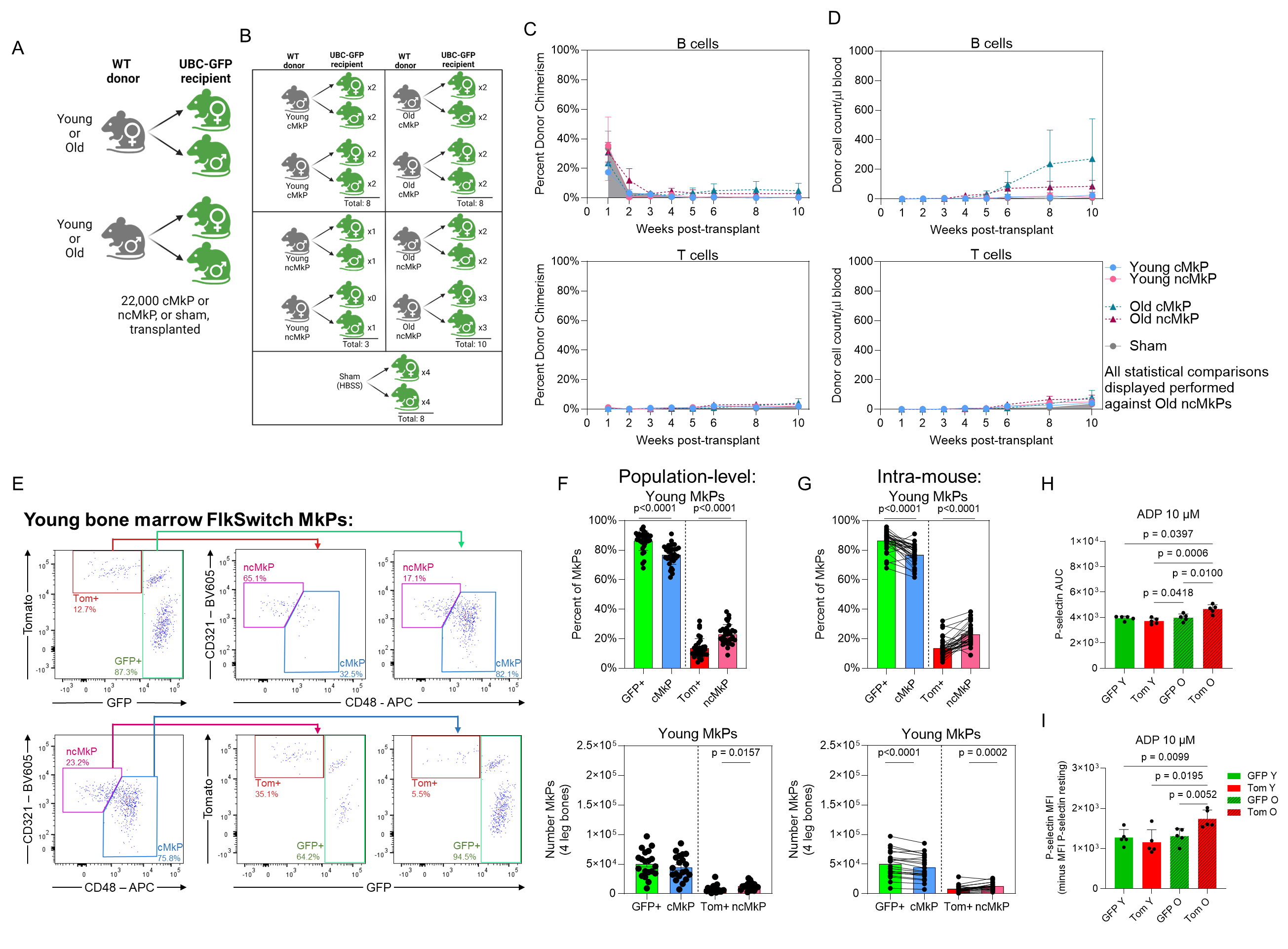


**Supplemental Figure 8. MkP transplantations and features of young FlkSwitch mice.**

**A-B.** MkP transplantation schematic and design.

**C-D.** The percent donor chimerism **C.** and donor-derived cell number **D**. of the indicated populations. n, experimental design, and statistics as in Figure 5E-F. Each point represents the mean ± standard error of the mean (SEM).

**E.** Example flow cytometry plots demonstrating the efficiency of using CD48 and CD321 in combination to enrich for young GFP+ and Tom+ MkPs among FlkSwitch mice.

**F-G.** Frequency and number of young MkPs defined as GFP+, Tom+, cMkP, or ncMkP. Unpaired or paired t-tests comparing GFP+ to cMkP and Tom+ to ncMkP to evaluate **F.** population-level and **G.** intra-mouse dynamics. Each point represents an individual mouse with lines connecting cells from the same mice. n=33 or 20 across more than 10 individual experiments.

**H-I.** Platelet activation from young and old mice with ADP (10 μM). P-selectin neo-exposure over time by **H.** the area under curve (AUC) and **I.** MFI of activated young (n=4) and old (n=5) FlkSwitch mice by ADP (10 μM). Statistical significance determined by one-way ANOVA with Tukey's multiple comparisons test.

**Supplemental Figure 9**

**
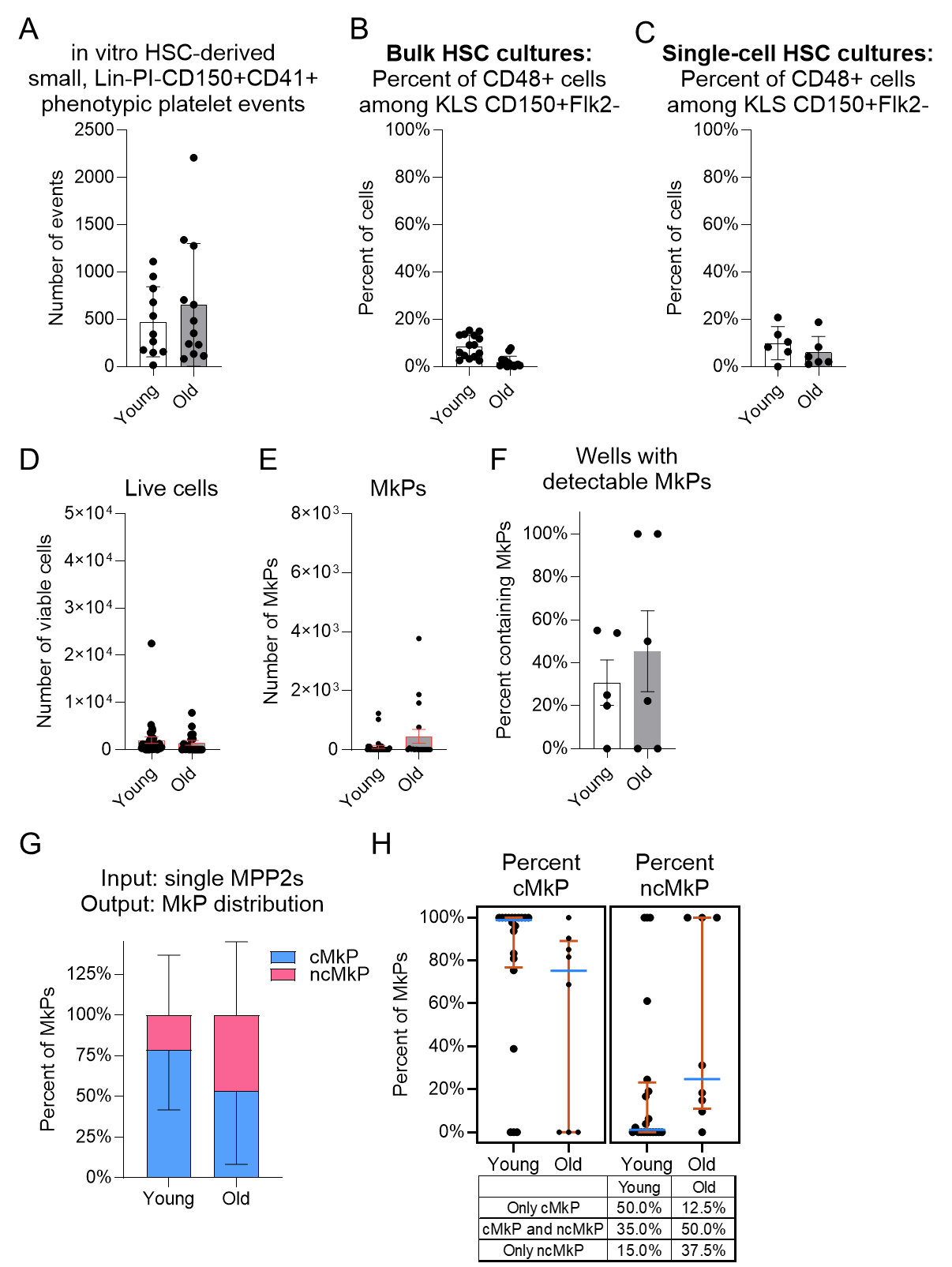
**

**Supplemental Figure 9. Dynamics of in vitro cultures.**

**A.** Flow cytometry events resembling platelets are detectable following seven days of bulk HSC culture. n=11 young and n=12 old.

**B-C.** Percentage of MPP2 cells among **B.** bulk or **C.** single-cell HSC cultures. n and number of experiments as in **B.** Figure 6A-E or **C.** Figure 7.

**D-H.** Retroactive index-based identification of wells initiated by MPP2s were identified and assessed in parallel with HSCs (see Figure 7). Young n=32 and old n=18 individual MPP2s from 5 individual mice across 3 individual experiments. D-H all statistically non-significant by two-tailed unpaired t-test.

**Supplemental Figure 10**

**
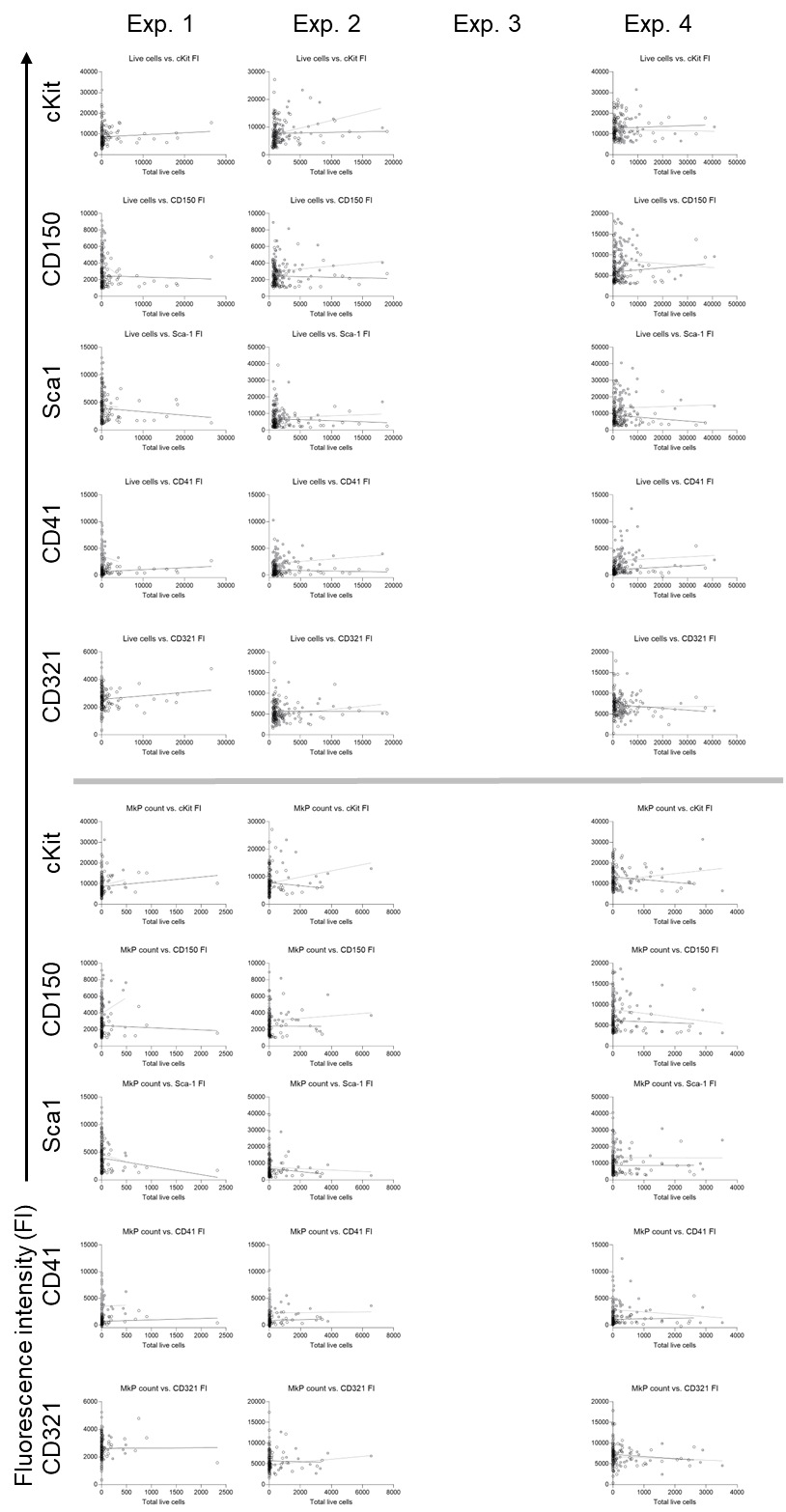
**

**Supplemental Figure 10 continued**

**
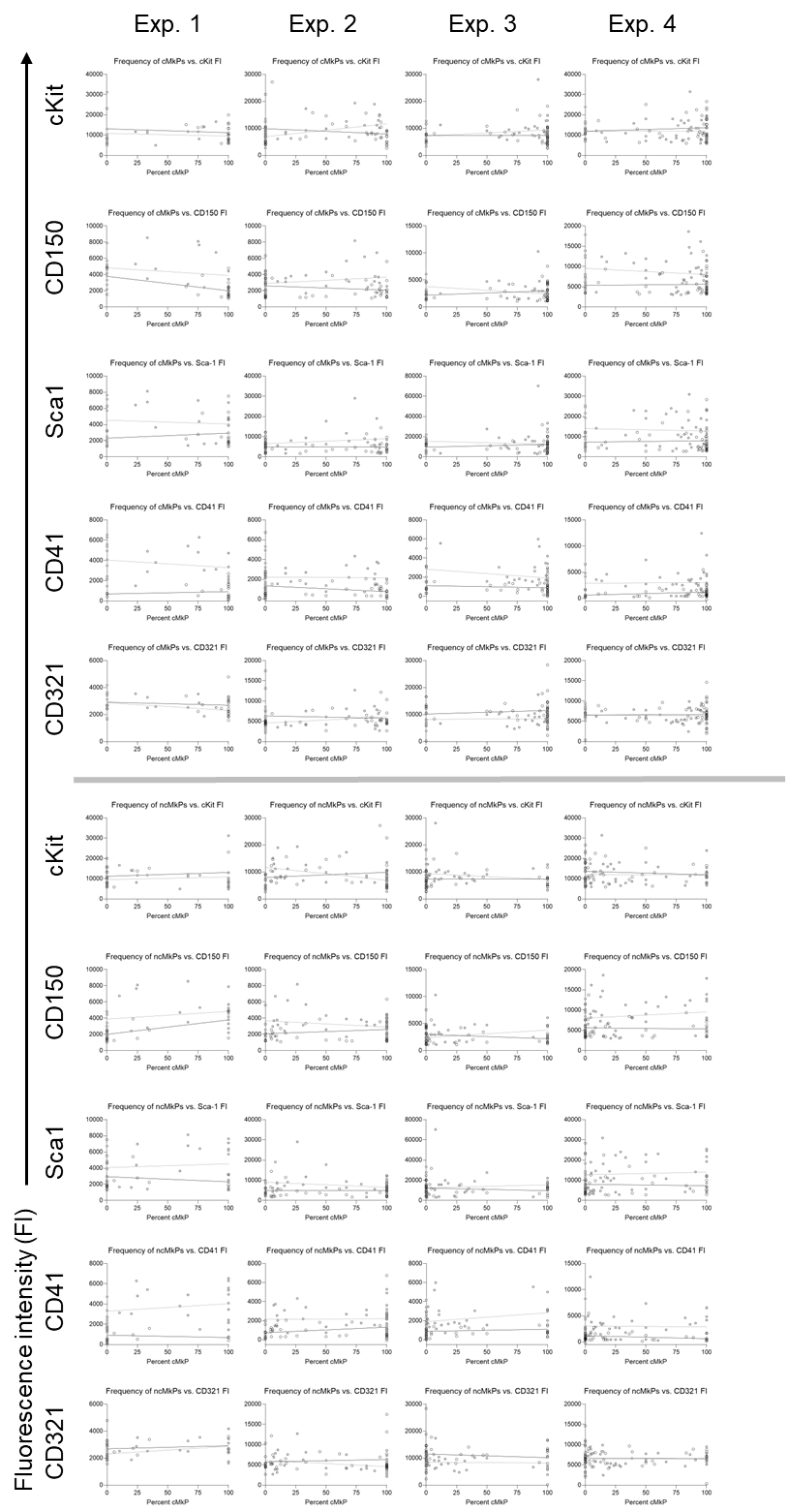
**

**Supplemental Figure 10. HSC phenotype does not correlate with MkP output in vitro.**

Flow cytometry single index sorted HSCs were correlated against their live cell and MkP output, comparing fluorescence intensity of each indicated marker against cell number or percent cMkP output across four experiments. Young are black open circles and trend line, whereas filled grey circles and trend line represent aged HSCs. See Table S4 for statistical report (Spearman two-tailed correlation). n and experimental design are as in Figure 7.

**Supplemental Figure 11**


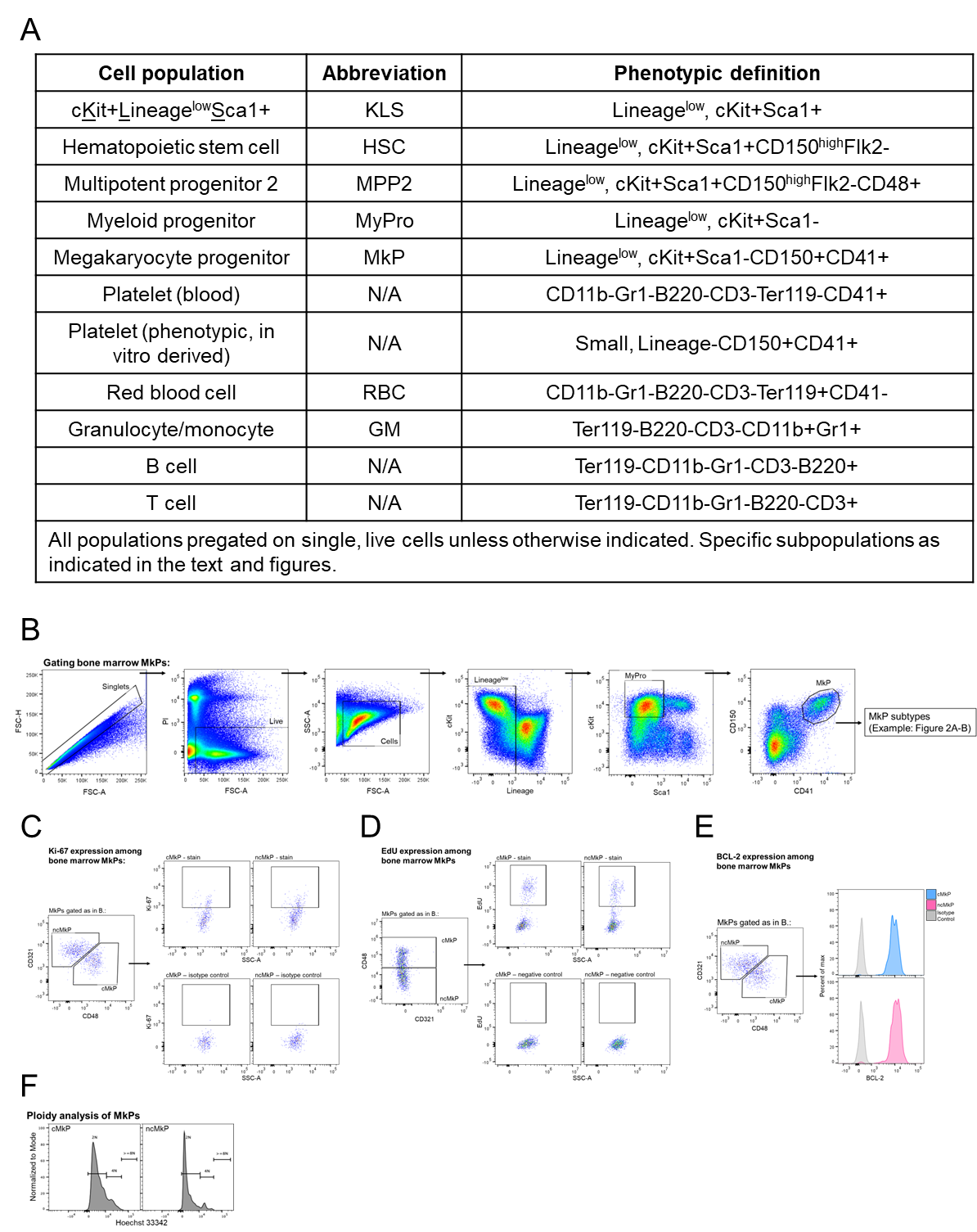


**Supplemental Figure 11 continued**


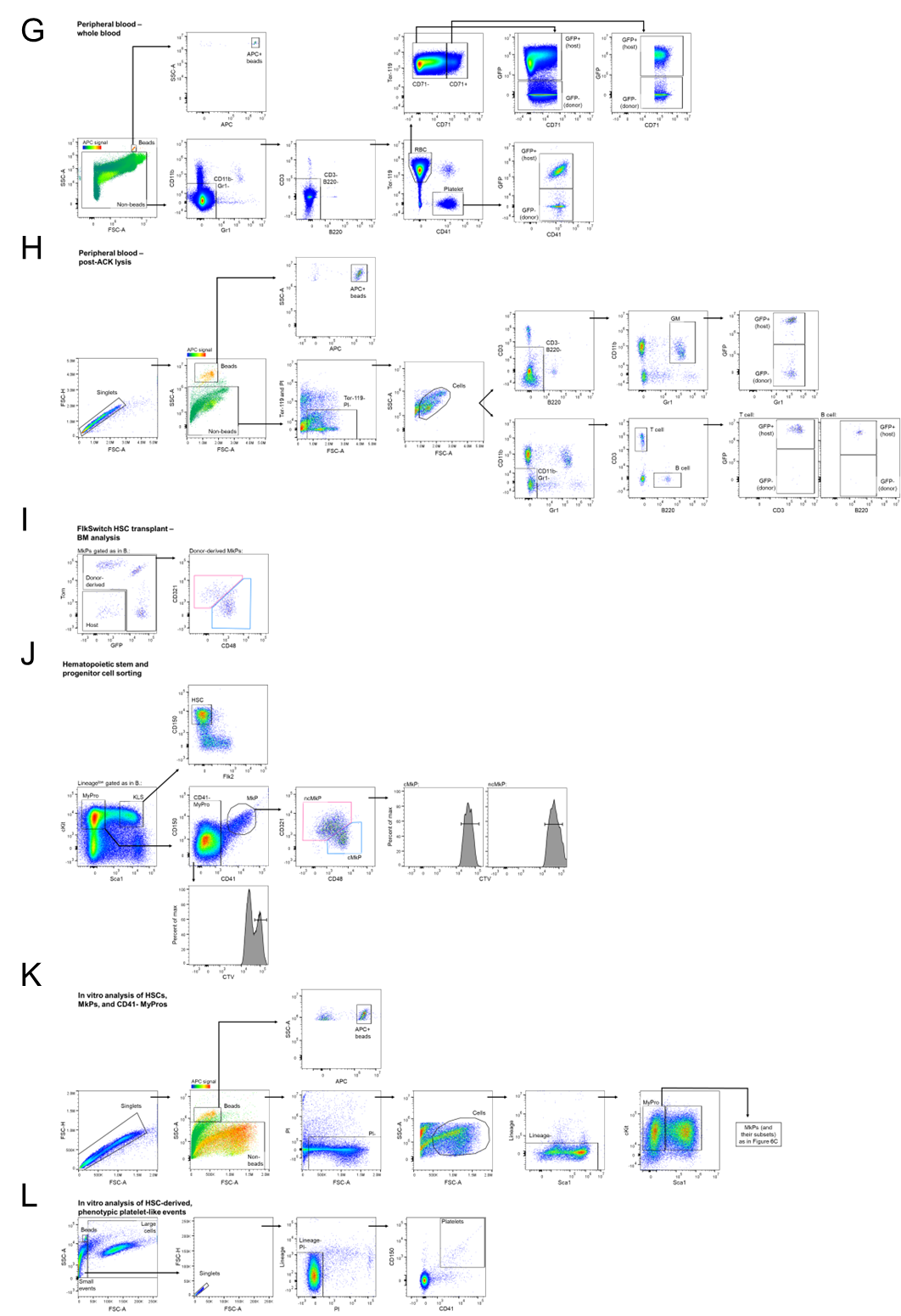


**Supplemental Figure 11. Phenotypic cell definitions and representative flow cytometry gating strategies.**

**A.** Table of all phenotypic cell definitions used.

**B.** Representative sequential flow cytometry gating strategy of BM MkPs.

**C.** Example intranuclear staining of BM MkPs for Ki-67 expression. The frequency of Ki-67+ cells (top row) for any given population was background subtracted using the isotype control (bottom row).

**D.** Assessment of EdU incorporation by BM MkPs. Not that the experimental procedure ablates CD321 expression, thus cMkPs and ncMkP defined by CD48 expression alone. The frequency of EdU+ cells (top row) for any given population was background subtracted using the negative control (bottom row).

**E.** Representative intranuclear assessment of BCL-2 among BM MkPs. The MFI of BCL-2 was background subtracted against the isotype control following standardized flow cytometry practices^3^.

**F.** Example ploidy assessment among BM MkPs.

**G-H.** Example sequential staining of peripheral blood, either as **G.** whole blood or **H.** following ACK lysis. Detection of APC+ Calibrite beads allows quantitation of numbers of cells/μl blood (see Methods). Determining donor chimerism of each population in MkP transplantations as shown in plots assessing GFP positivity.

**I.** Assessment of frequency and number of donor-derived cMkPs and ncMkPs following HSC transplantations.

**J.** Example FACS plots for HSCs, cMkPs, and ncMkPs. When in vitro CTV experiments were performed (see Methods), only uniform, high-expressing CTV+ cells were sorted as indicated.

**K.** Example in vitro analysis of HSCs, CD41- MyPros, or MkP subpopulations. Sequential staining and bead-based determination of cell number identical for all approaches.

**L.** Representative sequential gating for identification of phenotypic platelets following bulk in vitro HSC culture. Beads assessed as in **K.**

**Supplemental References**

1 Poscablo, D. M. *et al.* An age-progressive platelet differentiation path from hematopoietic stem cells causes exacerbated thrombosis. *Cell* **187**, 3090-3107 e3021, doi:10.1016/j.cell.2024.04.018 (2024).

2 Poscablo, D. M., Worthington, A. K., Smith-Berdan, S. & Forsberg, E. C. Megakaryocyte progenitor cell function is enhanced upon aging despite the functional decline of aged hematopoietic stem cells. *Stem Cell Reports* **16**, 1598-1613, doi:10.1016/j.stemcr.2021.04.016 (2021).

3 Manso, B. A. & Medina, K. L. Standardized flow-cytometry-based protocol to simultaneously measure transcription factor levels. *STAR Protoc* **2**, 100485, doi:10.1016/j.xpro.2021.100485 (2021).
